# Modeling an Excitable Biosynthetic Tissue with Inherent Variability for Paired Computational-Experimental Studies

**DOI:** 10.1371/journal.pcbi.1005342

**Published:** 2017-01-20

**Authors:** Tanmay A. Gokhale, Jong M. Kim, Robert D. Kirkton, Nenad Bursac, Craig S. Henriquez

**Affiliations:** 1 Department of Biomedical Engineering, Duke University, Durham, North Carolina, United States of America; 2 Medical Scientist Training Program, Duke University, Durham, North Carolina, United States of America; University of California San Diego, UNITED STATES

## Abstract

To understand how excitable tissues give rise to arrhythmias, it is crucially necessary to understand the electrical dynamics of cells in the context of their environment. Multicellular monolayer cultures have proven useful for investigating arrhythmias and other conduction anomalies, and because of their relatively simple structure, these constructs lend themselves to paired computational studies that often help elucidate mechanisms of the observed behavior. However, tissue cultures of cardiomyocyte monolayers currently require the use of neonatal cells with ionic properties that change rapidly during development and have thus been poorly characterized and modeled to date. Recently, Kirkton and Bursac demonstrated the ability to create biosynthetic excitable tissues from genetically engineered and immortalized HEK293 cells with well-characterized electrical properties and the ability to propagate action potentials. In this study, we developed and validated a computational model of these excitable HEK293 cells (called “Ex293” cells) using existing electrophysiological data and a genetic search algorithm. In order to reproduce not only the mean but also the variability of experimental observations, we examined what sources of variation were required in the computational model. Random cell-to-cell and inter-monolayer variation in both ionic conductances and tissue conductivity was necessary to explain the experimentally observed variability in action potential shape and macroscopic conduction, and the spatial organization of cell-to-cell conductance variation was found to not impact macroscopic behavior; the resulting model accurately reproduces both normal and drug-modified conduction behavior. The development of a computational Ex293 cell and tissue model provides a novel framework to perform paired computational-experimental studies to study normal and abnormal conduction in multidimensional excitable tissue, and the methodology of modeling variation can be applied to models of any excitable cell.

## Author Summary

One of the major challenges in trying to understand how arrhythmias can form in cardiac tissue is studying how the electrical activity of cardiac cells is affected by their surroundings. Current approaches have focused on studying cardiac cells *in vitro* and using computational models to elucidate the mechanisms behind experimental findings. However, tissue culture techniques are limited to working with neonatal, rather than adult, cells, and computational modeling of these cells has proven challenging. In this work, we have a developed a new approach for conducting paired experimental and computational studies by using a cell line engineered with the minimum machinery for excitability, and a computational model derived and validated directly from this cell line. In order to create a model that reproduces the diversity, rather than simply the average behavior, of experimental studies, we have incorporated a simple yet novel method of inherent variability, and explored what types of experimental variation must be incorporated into the model to recapitulate experimental findings. Using this new platform for paired experimental-computational studies with inherent variability, we will be able to study and better understand how changes in cardiac structure such as fibrosis and heterogeneity lead to conduction slowing, conduction failure, and arrhythmogenesis.

## Introduction

One of the major challenges in the field of cardiac electrophysiology is quantifying the electrical dynamics of myocytes in the context of their environment. The electrical activity of cardiomyocytes *in vivo* is modulated by the other excitable and unexcitable cells to which they are coupled, as well as the complex interstitial space in which they are embedded. Making multisite measurements of the transmembrane potential is technically difficult to perform *in situ* and hence limited information is available to characterize the cells’ complex response to stimuli and drugs. One approach to studying excitable cells in context is to develop detailed *in silico* computational models of isolated excitable cells and tissues, and to use these models to infer the behavior of the real cells on which they are based. In most cases, computational membrane models are derived from experimental data obtained using various patch clamp techniques, often performed in different labs, under different conditions and often in cells from different species [1]. Moreover, because the details of the complex native tissue environment are poorly understood, most computational tissue models make use of significantly simplified representations of the native 3D tissue structure.

An alternative approach for studying cells’ electrical dynamics in context with other cells is to use *in vitro* cell cultures. While typically limited to two dimensions and lacking a defined interstitial space, cultured cell monolayers can reproduce many features of the natural tissue through the manipulation of cell orientation, spacing and shape [2–5]; engineered monolayers have previously been used to study complex phenomenon such as conduction block, re-entry and spiral wave formation in 2D [6]. At present, these methods are limited to the use of neonatal cells as culturing of adult cardiac cells into confluent, electrically coupled monolayers has proven difficult. Unfortunately, the intrinsic currents of neonatal cells have been difficult to model as they change rapidly through development. As a result, there is currently no robust and tractable framework for the careful comparison of computational predictions with biologically analogous experimental measurements in multidimensional tissue.

Kirkton and Bursac recently demonstrated the ability to genetically engineer a synthetic excitable cell line through the addition of only two ion channels (Na_v_1.5 and Kir2.1) into the immortalized and non-excitable HEK293 cell line. They further were able to electrically enhance the intercellular connectivity of these excitable HEK293 cells (named Excitable-293 or Ex293 cells) by overexpressing connexin-43 gap junctions to form an excitable, engineered monolayer capable of propagating action potentials [7]. These excitable monolayers were subsequently used to study a wide range of electrophysiological behaviors such as reentry and conduction failure [8,9]. The limited number of ionic currents in these novel biological constructs as well as their relative stability over time (compared to maturing neonatal cells) suggest the strong potential to be modeled computationally with high fidelity.

Despite their monoclonal origin and relatively simple electrophysiology compared to adult cardiomyocytes, Ex293 cells and tissues exhibit moderate variability in their action potential characteristics (e.g. action potential duration, maximum upstroke velocity, etc) and conduction properties [7,9]. This observed variability results from a combination of beat-to-beat variability in single cells, cell-to-cell variability within a monolayer, and variability between different tissue-cultured monolayers. While biological variability has a relatively moderate impact on macroscopic conduction under well-coupled normal conditions [10–12], it will likely play a more significant role in experimental scenarios replicating disease states such as fibrosis, cellular uncoupling, reduced excitability, and premature stimuli [13]. As such, it is important to consider intrinsic variability when developing computational membrane models that are intended for the study of complex conduction behavior. In general, most computational models are constructed using mean experimental-derived properties, and efforts at modeling variability have been focused on regional differences (epicardial vs endocardial, or apex vs base) or on variability between isolated cells [14,15]; the concept of simulating tissues with variable membrane properties has only recently been explored [12].

In this study, we expanded on the work of Kirkton and Bursac [7] by using published experimental single cell and monolayer data to develop a computational model of the Ex293 cell that can be used for future paired experimental-computational studies. In doing so, we examined what sources of variability were required in the model of this highly-simplified excitable cell in order to reproduce experimental behavior. Our results show that the incorporation of cell-to-cell and inter-monolayer ionic conductance variation as well as inter-monolayer conductivity variation was necessary to reproduce the behavior of propagating action potentials in Ex293 monolayers over a range of experimental conditions. Moreover, we demonstrate that non-random spatial organization of cell-to-cell variation does not significantly affect macroscopic conduction, indicating that random spatial distribution of cell-to-cell ionic variation can adequately capture the impact of experimental ionic variability.

## Results

### Development of the Base Membrane Model

**Fig 1:**
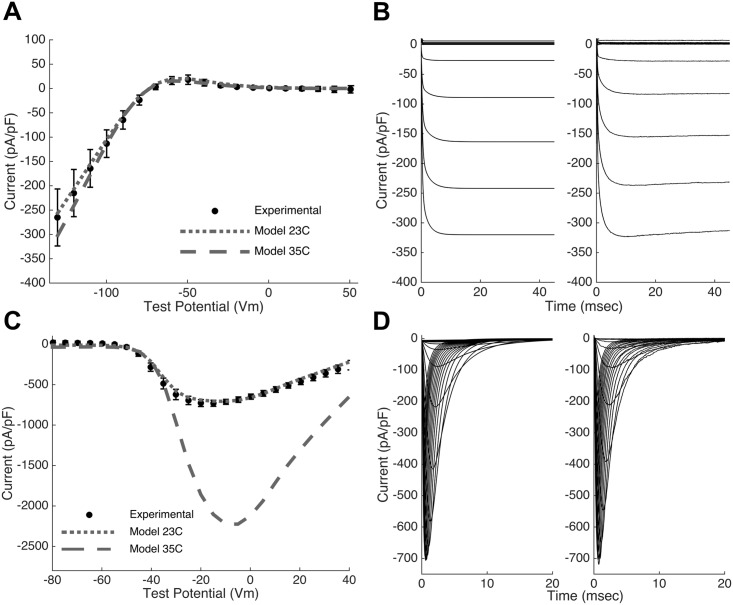
Model recapitulates experimental current properties. (A,C) The Ex293 membrane model replicates (dotted line) the experimentally observed peak current-voltage relationships (closed circles) of the transfected potassium and sodium channels at 23°C. An increase in current density and shift in voltage dependence is seen in the model at physiological temperature (dashed line) (B,D) The model (left panel) also replicates the dynamics of channel activity (right panel). Note that the model conductances in panels B and D were selected to match the experimental traces; these are not the same as the mean model conductances.

The Ex293 membrane model includes four constitutive currents: the inward rectifying potassium current (I_K1_, carried by the Kir2.1 channel) and the fast voltage-gated sodium current (I_Na_, carried by the Na_v_1.5 channel), both of which are transfected into HEK293 cells to create the Ex293 line; as well as two endogenous HEK293 currents, a voltage gated sodium current [16] and a delayed-rectifier potassium current [17–19]. As described in *Methods*, mathematical descriptions of each current were formulated directly from previously reported mean experimental data from Kirkton and Bursac [7] and others [16,17]. The inward rectifying potassium current was modeled using a single activation gate and the resulting model is able to reproduce the current-voltage curve for the peak K^+^ current (Fig 1A, dotted line) as well as the time courses of potassium current elicited by a step change in membrane potential at room temperature (Fig 1B). The transfected voltage gated sodium current was modeled with three identical activation gates, and one inactivation gate with fast and slow components. At room temperature (23°C), the model is able to recapitulate the experimentally observed current-voltage curve for the peak Na^+^ current (Fig 1C, dotted line) as well as the dynamics of the sodium current in response to step changes in membrane potential (Fig 1D).

### Fitting of the Membrane Model

**Fig 2:**
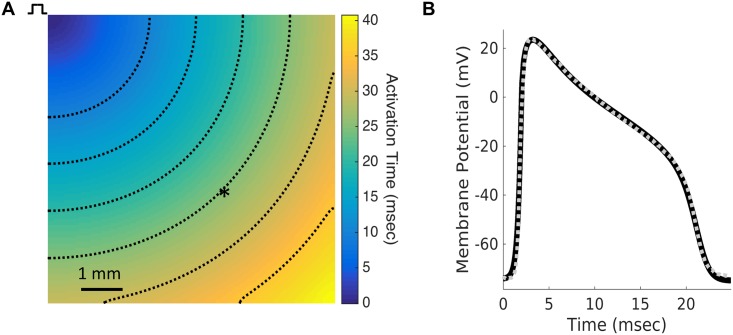
Model action potential replicates experimental action potential. (A) Model fitting was performed by simulating conduction in a 2-D monolayer and recording an action potential 6 mm from the stimulus site (asterisk). Dashed lines are isochrones of activation at intervals of 5 ms. (B) The action potential generated by the fitted Ex293 membrane model (solid black line) replicates the morphology of the experimentally-recorded Ex293 action potential from [7] (dashed gray line).

Because the published electrophysiological data provides insufficient information to fully specify the Ex293 membrane model at 35°C, a multiobjective genetic search algorithm was used to determine optimal values for several free parameters in order to match experimentally observed conduction properties. Eight free parameters were fit using the genetic search technique, including the maximal current densities for each of the four currents and the bulk tissue conductivity (see Table 1 for all fitted parameters). For each trial parameter set, conduction was simulated in a two dimensional continuous monodomain (Fig 2A) using the Cardiowave system [20], and two error functions: (1) the root mean square error of the simulated action potential compared to a representative experimental action potential, and (2) the absolute error in simulated conduction velocity (CV) were calculated. The genetic algorithm was executed nine times, yielding different solutions, and the parameter set from each Pareto front that minimized the mean square action potential error was selected.

**Table 1:** Model optimization via genetic search algorithm (n = 9 runs).

Model parameter	Mean (SD)	Units	Search Range
G_Na_	90.76 (0.77)	mS/cm^2^	0–200
G_K_	6.623 (0.11)	mS/cm^2^	0–30
G_Na, wt_	0.645 (0.052)	mS/cm^2^	0–2
G_K, wt_	0.147 (0.022)	mS/cm^2^	0–1
V_1/2_ for b_∞_ (I_K,wt_ activation)	16.29 (7.14)	mV	-20–40
*k* for b_∞_ (I_K,wt_ activation)	34.80 (4.44)	mV	10–40
τ_b_ (I_K,wt_ time constant)	0.56 (0.04)	ms	0.01–1.0
σ (Bulk tissue conductivity)	1.143 (0.014)	mS/cm	0–2
Root mean square error	1.11 (0.211)	mV	
Absolute CV error	0.068 (0.060)	cm/s	
Results using mean parameter estimates		Units	
Root mean square error	1.66	mV	
Absolute CV error	0.116	cm/s	
Results using selected parameter estimates		Units	
Root mean square error	1.109	mV	
Absolute CV error	0.0001	cm/s	

Each run of the genetic algorithm required an average of 112 generations to converge; the mean parameter values identified by the genetic algorithm across multiple runs are shown in Table 1. While the genetic algorithm searched a large parameter space, multiple runs resulted in parameter estimates with relatively little variability, indicating a strong likelihood that the fit results recreate the biological behavior. The parameter set that provided the lowest mean square error of action potential fit was used in the 35°C model. Fig 2B shows that action potential generated by these parameter values reproduces the representative experimentally recorded action potential with a high degree of accuracy, with a root-mean-square error of 1.109 mV and a CV error of 0.0001 cm/s. In addition, the simulated action potential replicates several other metrics characterizing experimental action potentials obtained from multiple cells (S1 Table, Columns 1 and 2).

Based on the genetic algorithm fits, the change in temperature from 23°C to 35°C resulted in an approximately 3-fold increase in the I_Na_ maximum conductance, and a 50% increase in the I_K1_ maximum conductance, which is in line with previous studies [21–23]. The total temperature-induced changes in the current-voltage relationships of the I_Na_ and I_K1_ currents, due to both temperature-dependent conductance changes (as determined by the genetic algorithm fits) and temperature-dependent shifts in activation and inactivation (as determined based on previous studies), are shown in Fig 1A and 1C (dashed lines). The model qualitatively matches the net ionic current recorded experimentally during single-cell action potential (AP) clamp recordings at 23°C (S1A and S1C Fig). At 35°C, the model shows temperature induced changes in ionic currents (S1D Fig) including a substantial increase in inward sodium current. Examination of the role of the individual currents shows that the I_Na_ current is responsible for rapid depolarization while the I_K1_ current resists depolarization and is responsible for rapid repolarization. In addition, the model suggests that an endogenous HEK293 potassium current plays an important role in the repolarization of the Ex293 cell as its low outward current gives the plateau phase of the action potential its shape, and lowers the membrane potential from peak voltage (~ 20 mV) to a voltage where the transfected I_K1_ current activates and initiates the rapid depolarization phase. In contrast, a relatively small endogenous sodium current within HEK293 cells appears to only play a minor role in the Ex293 action potential.

### Addition of Variation to the Base Model

**Fig 3:**
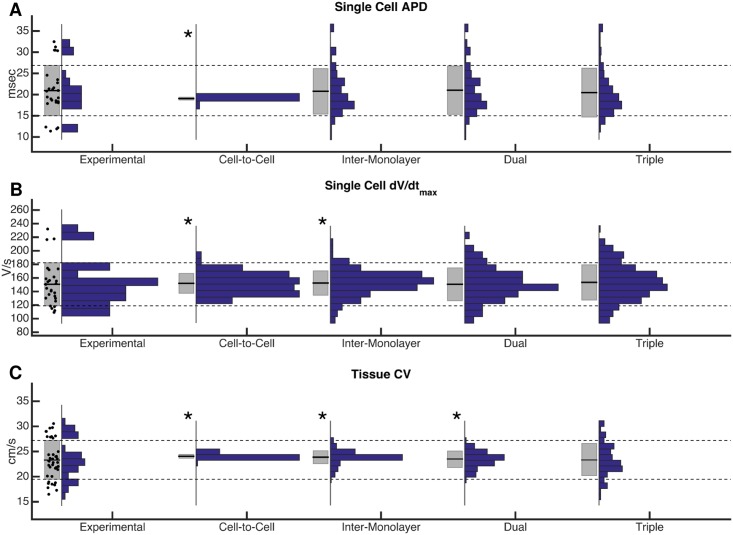
Comparison of methods of modeling variability. Modeling of cell-to-cell conductance variation and inter-monolayer conductance variation alone, or in combination (“dual variation”) is not sufficient to match all experimental variability. The addition of inter-monolayer bulk conductivity variation (“triple variation”) allowed for the replication of experimentally observed variability in single cells (A and B), as well as in macroscopic conduction velocity (C). Box plots to the left of each histogram indicate mean +/- one standard deviation. Asterisks indicate that variances are significantly different (p < 0.05) from experimental variability, using Levene’s test for equal variances.

To replicate the observed experimental variability in action potential and conduction properties, variation of ionic conductances and of tissue conductivity was incorporated into the model by scaling model parameters by a factor randomly selected from a normal distribution with a mean of one and a specified standard deviation. Action potential shape properties were measured from a single location in the monolayer (i.e. a single node in the computational grid), to compare to experimental data from sharp intracellular electrode recordings; conduction properties were measured by simulated optical mapping (see Methods) of 2-D computational monolayers. Experimentally observed variability in [7] was attributed either entirely to cell-to-cell variation (each cell has slightly different properties), entirely to inter-monolayer variation (each monolayer has slightly different properties), or to a combination of both types of variation. The addition of cell-to-cell ionic conductance variation to the model with standard deviations of variation as high as 0.50 led to a small degree of variability in single cell maximum upstroke velocity that was insufficient to match that seen experimentally (variances unequal by Levene’s test, p < 0.01) and almost no variability in either single cell action potential duration (APD) or macroscopic monolayer CV (Fig 3, Column 2). Cell-to-cell variation was then eliminated and all experimentally observed variability was instead modeled as due to inter-monolayer current variation. Random normal variation of the ionic conductances for each monolayer with a standard deviation of 0.125 approximately replicated variability in single cell APD; however, insufficient variability was seen in mean monolayer CV (p < 0.01), and the lack of cell-to-cell variation resulted in insufficient variability in maximum upstroke velocity compared to experimental observations (p < 0.05) (Fig 3, Column 3)

**Fig 4:**
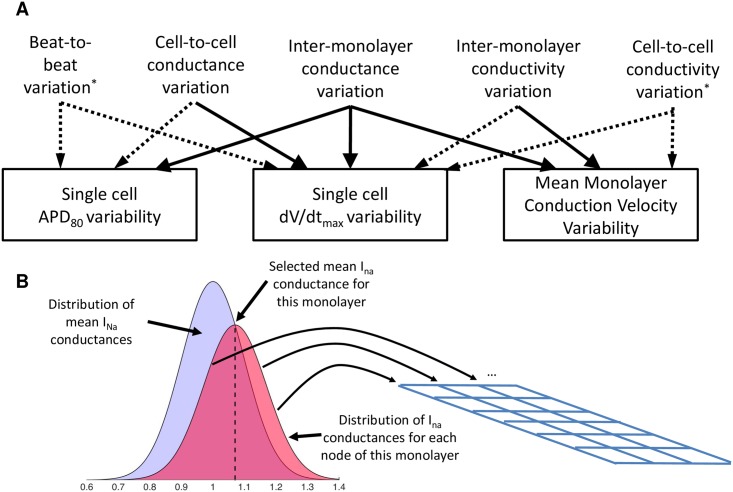
Modeling variability. (A) Types of variation and their relative impacts on measured properties. Significant linkages are shown with solid lines while weak effects are shown with dashed lines. Types of variation marked with an asterisk are not described in depth but are included for completeness (B) In order to model both cell-to-cell and inter-monolayer conductance variability, a mean monolayer conductance (black dashed line) is selected from a random normal distribution (blue distribution). The conductance of each node within the monolayer is then selected from a random normal distribution around the mean monolayer conductance (red distribution)

A combination of inter-monolayer conductance variation and cell-to-cell conductance variation (termed “dual variation”) was explored. Random normal variation with standard deviation of 0.125 was used to select each monolayer’s mean conductances, and within each monolayer, further random normal variation with standard deviation of 0.125 was used to select ion channel conductances of individual tissue nodes (Fig 4B). The resulting conductances, when pooled across multiple monolayers, were normally distributed a coefficient of variation of 0.177, comparable to that reported for current density in isolated cells (0.22 for I_K_ in Ex293 [7], 0.13 for the endogenous potassium current in HEK293 [17]), and well within the range of intraclonal protein expression variation seen in monoclonal cell lines [24]. This formulation was found to result in simulated variability that approximately matched experimentally observed single cell upstroke velocity variability, as well as experimental APD variability; however, dual variation failed to capture the degree of variability seen in the mean monolayer CV (p < 0.05) (Fig 3, Column 4). Inter-monolayer conductivity variation (i.e., variation of bulk tissue conductivity for each monolayer) was then added to the dual variation model to yield “triple variation”. Each monolayer’s tissue conductivity was perturbed with random normal variation with standard deviation of 0.25. Ultimately, this combination of model parameter variation was able to faithfully replicate the degree of variability observed experimentally in macroscopic conduction properties (mean CV and APD) as well as the variability in experimental single cell action potential properties (APD and maximum upstroke velocity) (Fig 3, Column 5; and S1 Table). The effects of each type of variation on measured variability are summarized on Fig 4A. While cell-to-cell variability is likely to remain constant between experimental studies, the degree of experimental inter-monolayer variability, which depends on culture conditions, initial cell seeding and monolayer confluence, will vary between experimental preparations. Indeed, a moderate decrease in mean monolayer APD and CV variability during 2Hz pacing was noted between experimental results in [7] and later studies in [9]. A 50% reduction in the inter-monolayer variability of ionic conductances and tissue conductivity was necessary to match the experimental variability noted observed in [9].

A sensitivity analysis was performed to understand how variation in each of the Ex293 conductances (see Eqs 3, 5, 7 and 8 and S2 Table) affect macroscopic conduction properties, and to ensure that small perturbations in model parameters led to physiologically reasonable behavior. Maximum channel conductances in monolayers with no variability were scaled from 0.5 to 1.5-fold, individually. Perturbations in the exogenous sodium current (I_Na_) had a substantial, non-linear effect on CV and APD (S3 Fig), with a 40% decrease in conductance leading to a 35% decrease in CV and 24% decrease in APD, while a 40% increase in conductance led to 17% conduction speeding and a minimal increase (6%) in APD. The effect of perturbations in the exogenous potassium current (I_K_) on CV was nearly linear (R^2^ = 0.995) with a slope of -0.17 (% change in CV / % change in conductance), while the effects on APD were drastically non-linear (40% increase and decrease in conductance lead to 25% decrease and 79% increase in APD, respectively). The effect on APD is consistent with the dominant role of the I_Na_ and I_K1_ currents in the upstroke and downstroke of the action potential, respectively. In addition, during the action potential upstroke, the inward I_Na_ current that causes depolarization is opposed by the outward I_K1_ current, which drives the membrane potential towards rest; the effects of perturbations in I_Na_ and I_K1_ on CV are consistent with the roles of these currents during the action potential upstroke. In contrast with the effects of perturbation of the exogenous currents, perturbations in endogenous currents (I_K,wt_ and I_Na,wt_) substantially affected APD without significantly altering CV. This is consistent with the activity of the I_K,wt_ and I_Na,wt_ currents in the plateau and early repolarization of the action potential and the lack of activity during the depolarization and late repolarization.

### Evaluation of Dynamic Properties

**Fig 5:**
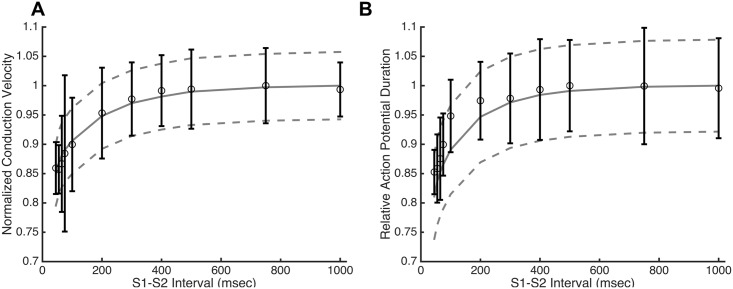
Ex293 restitution behavior. The base model (solid line) is able to closely mimic the experimentally observed (open circles) conduction velocity (A) and action potential duration (B) restitution profiles (R^2^ = 0.97 and 0.82, respectively). Model variability (dashed lines represent +/- 1 SD) approximates the degree of experimental variability. Note that experimental data from [9] is plotted as mean ± s.d.

The Ex293 model was validated over a range of experimental conditions that had been studied previously [7–9]. For example, Kirkton and Bursac measured the CV and APD restitution by examining the response of a monolayer to a premature stimulus delivered following pacing of the monolayer at a constant rate. The restitution properties were obtained in the model by stimulating a strip of tissue at 2Hz (S1) and applying a premature stimulus (S2) at incrementally earlier times. Fig 5A shows that baseline mean Ex293 model is able to reasonably reproduce the experimentally observed CV restitution (Fig 5A) and APD restitution (Fig 5B) curves, with R^2^ values of 0.97 and 0.82, respectively. The significant variability noted in experimental APD restitution curves [9] is approximately replicated by the inclusion of cell-to-cell and inter-monolayer variability in the Ex293 model (Fig 5, dashed lines represent one SD above and below the mean).

**Fig 6:**
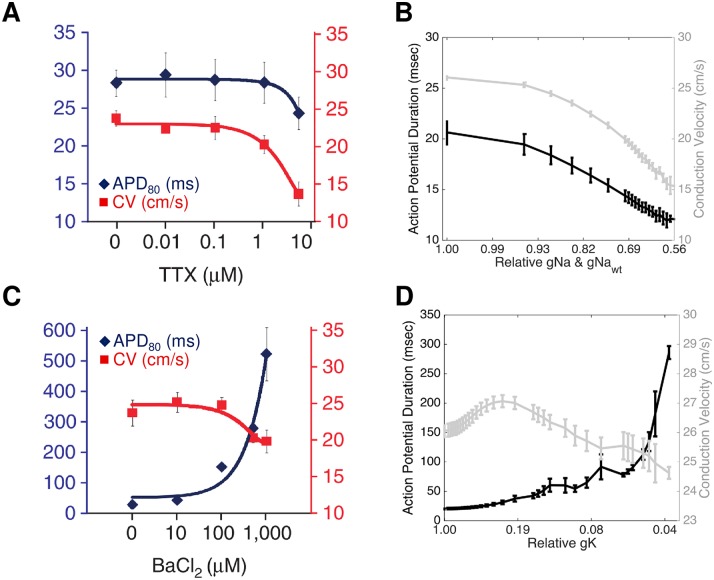
Comparison of simulated channel blockade with experimental findings. Simulated blockade of the sodium currents via TTX (B) and of IK_1_ current via barium chloride (D) qualitatively replicates the effects of experimental blockade (A: experimental TTX; C: experimental BaCl_2_). Note that there is a sigmoidal relationship between drug dose and degree of block, and that the x-axis of panels B and D has been inverse-sigmoidally transformed to allow for direct comparison of simulated response and experimental results. Panels A and C from [7], used with permission.

Kirkton and Bursac also explored the effects of the ion channel blockers tetrodotoxin (TTX) and barium chloride (BaCl_2_) on the Ex293 cells. Because both the exogenous and endogenous sodium currents are sensitive to TTX [7,16], simultaneous perturbation of both sodium channels is analogous to application of TTX in an experimental preparation. TTX blocks sodium current in a dose dependent but not voltage sensitive manner [25]. While suppression of the sodium currents in the model cannot be correlated with a specific TTX dose in the absence of an Ex293 TTX dose-response curve, the effect of simulated sodium channel blockade (Fig 6B) is qualitatively similar to the response of Ex293 monolayers to TTX in Kirkton and Bursac (Fig 6A) [7], with increasing sodium block leading to accelerating decreases in CV and APD until conduction failed when sodium conductance was reduced by more than 45% (Fig 6B).

Barium chloride acts as a blocker of some potassium channels: while the transfected Kir2.1 potassium channels are sensitive to barium chloride [7], the endogenous potassium currents in HEK-293 cells are not affected [26]; therefore, reductions in the transfected potassium conductance are analogous to treatment of Ex293 cells with BaCl_2_. However, barium chloride blocks potassium current not only in a dose-dependent manner, but also in a voltage-sensitive manner [27–29]. Based on data from published figures from [27–29], a description of barium chloride block was incorporated that reflects the variation in block as a function of membrane potential (S4 Fig). Using this model, our simulated Ex293 monolayers behave similarly to experimental monolayers when exposed to barium chloride (Fig 6C and 6D). As the potassium current is blocked, there is an increase in CV up to 27.1 cm/s due to decreased outward current (i.e., that opposes depolarizing inward sodium current) during the upstroke of the action potential. When the potassium current at low membrane potentials is decreased by more than 75% (corresponding to a decrease by 26% at 0 mV), conduction slowing occurs, due to sodium channel inactivation as the resting potential rises. Simulated barium chloride-induced reduction in potassium current also results in a up to 20-fold monotonic, exponential increase in APD, as shown by Kirkton and Bursac [7].

**Fig 7:**
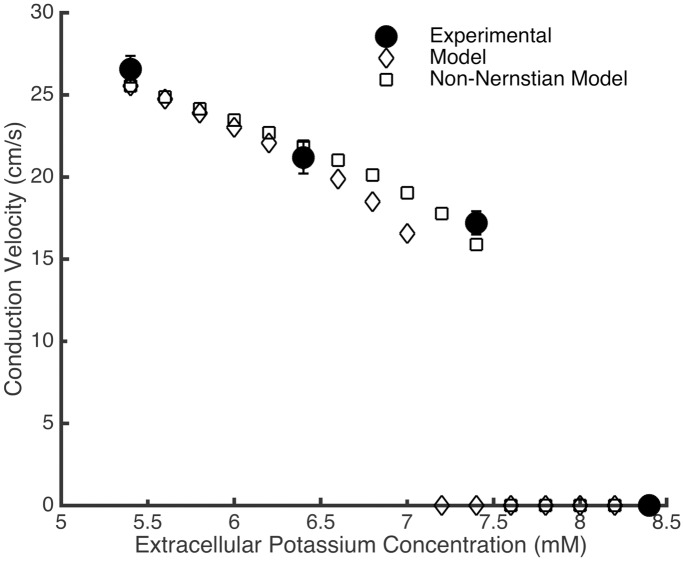
Conduction slowing due to increased extracellular potassium. An increase in extracellular potassium concentration in experimental Ex293 monolayers leads to conduction slowing, and conduction failure for concentrations greater than 7.4 mM (closed circles). The model shows similar behavior, but conduction failure occurs at a lower concentration (open diamonds). When the model is modified to reflect non-Nernstian changes in potassium reversal potential, conduction slowing more closely replicates experimental observations (open squares).

Finally, because the I_K1_ conductance is dependent on extracellular potassium concentration ([K_o_]), the effect of varying this concentration was also examined. While neonatal rat ventricular myocytes require a 12 mM increase in extracellular potassium before conduction fails, experimental Ex293 monolayers experience conduction block when extracellular potassium is increased by as little as 2 mM from 5.4 mM to 7.4 mM [30]. In our simulated Ex293 monolayers, increases in extracellular potassium result in conduction slowing by up to 35.2% with a 1.6 mM increase in [K_o_]; any further increase in extracellular potassium results in a failure to fire and propagate action potentials (Fig 7). This effect is due to a combination of increased I_K1_ conductance [31,32]; changes in the I_K1_ driving force due to an raised potassium reversal potential; and sodium channel inactivation due to elevation of the resting membrane potential. The threshold for causing conduction failure is lower than that observed experimentally. However, when the model is adjusted such that the change in resting membrane potential due to changes in extracellular potassium are not perfectly Nernstian (based on data from Bailly et al. [32], using a change in reversal potential of 51 mV per decade change in [K^+^]_o_, rather than 58 mV as predicted by the Nernst equation), the extracellular potassium concentration must be increased by at least 2.2 mM to trigger conduction failure, comparable to the threshold observed experimentally.

### Examining Spatial Organization of Variation

Because monolayers and *in vivo* tissues arise from a smaller number of parent cells that divide and grow to confluence, it is possible that cell-to-cell level variability is spatially organized rather than randomly distributed. The impact of this spatial organization of variation on macroscopic conduction was examined using an idealized scenario with a central region of prolonged APD (due to reduced mean I_K1_ conductance) and reduced cell-to-cell variation. The distribution of ionic properties in the remainder of the monolayer was adjusted such that the overall distribution of I_K1_ conductance was not altered.

**Fig 8:**
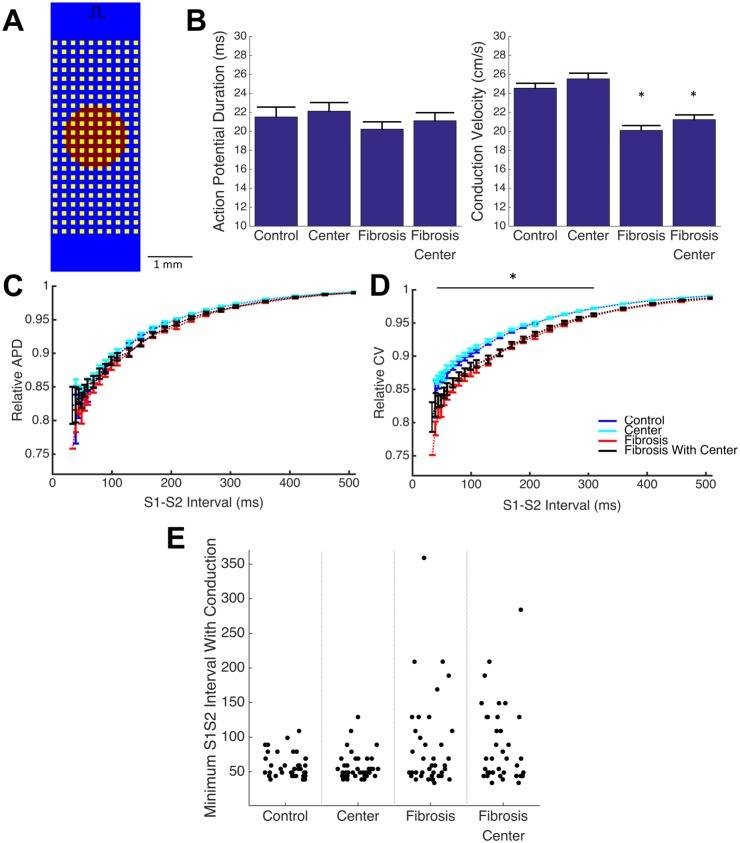
Spatial organization of ionic variation does not affect macroscopic conduction. The introduction of a central region with reduced variance and prolonged APD (A, red) into a tissue model with and without non-conductive fibrosis-like obstacles (A. yellow) does not cause additional conduction slowing and APD shortening at 1 Hz pacing beyond the effect of fibrosis alone (B). A fibrosis induced exaggeration of CV slowing (D), but not APD shortening (C), at short diastolic (S1-S2) intervals (plotted as mean +/- standard error) is also unaffected by the spatial organization of variation. In addition, spatial organization maintains but does not enhance premature failure, as characterized by minimum S1-S2 intervals able to fully conduct across the domain (E). (* p < 0.05 main effect of fibrosis)

The addition of the central region of prolonged APD and reduced variance does not significantly affect APD or CV during 1Hz pacing (Fig 8B). In addition, no statistically significant change in restitution behavior or in minimum viable S1-S2 interval were observed (Fig 8C–8E). Because it is well established that cell-to-cell variation is masked by strong coupling of the tissue, we examined how spatial organization of variation affected macroscopic conduction in tissue with functional decoupling via simulated, idealized fibrosis.

Fibrosis was simulated via the addition of a regular field of non-conductive obstacles (Fig 8A), which results in a 18.1% reduction of mean CV and 6.0% reduction in mean APD during 1 Hz pacing (Fig 8B), as well as exaggerated conduction slowing at shortened diastolic intervals below 300 ms when compared to non-fibrotic tissues (normalized to 1Hz CV) (Fig 8D). In addition, the tissue with regular fibrosis-like obstacles exhibits increased variability in conduction failure behavior compared to the control tissue: only 2.5% monolayers fail to conduct at an S1-S2 interval longer than 100 ms in the control case, while in the fibrotic monolayer, 25% fail at S1-S2 intervals greater than 100 ms (p < 0.05).

The addition of the central region with reduced variation and prolonged APD to the fibrotic tissue results in no significant change in macroscopic conduction behavior at 1Hz or in behavior at shortened diastolic intervals, beyond those observed in the fibrotic tissue alone (Fig 8B, 8C and 8D). Further, the addition of a central region of prolonged APD and reduced variance does not further increase failure variability (30% failure rate at S1-S2 interval greater than 100 ms vs 25% in fibrotic tissue without central region).

## Discussion

The goal of this work was the develop a mathematical model that allows for paired computational-experimental studies using a simple, synthetic excitable Ex293 cell, and to examine what types of variability were necessary in the model to replicate experimentally observed behaviors in both normal and abnormal conduction conditions. We modeled individual ionic currents using Hodgkin-Huxley formulations and used a parallelized multiobjective genetic search algorithm to identify unknown model parameters to recreate experimental action potential traces. To capture the variability in the electrical properties of the cultured Ex293 cells, we added random variation to current and tissue properties to replicate the degree of electrical variability observed experimentally, and we validated the resulting model across a series of test conditions. Our model captures both the mean behavior and the experimental variability of the engineered cells *in vitro* over a range of conditions, opening the possibility of using this novel computational/experimental framework to explore mechanisms of conduction failure under a variety of conditions.

### Developing Ex293 Membrane Model

Various groups have reported extensively on the presence of endogenous potassium ([17–19,33–35]), sodium ([16,36]), calcium ([37,38]) and chloride ([34]) currents in HEK293 cells [39]. In our model, we chose to include two of these currents: the endogenous potassium current, which has been implicated in allowing spiking behavior in HEK293 cells expressing exogenous sodium channels [40]; and the endogenous sodium currents of relatively large magnitude whose presence would affect plateau behavior of the action potential. An in-depth discussion of the description of individual membrane currents is presented in Supplementary Text 1. We have shown that Hodgkin-Huxley style currents definitions, as built using published literature current descriptions and adapted to physiological temperatures, are sufficient to faithfully reproduce both the single-current (Fig 1) and whole-cell properties (S1 Fig) seen in experimental recordings.

In our studies, the membrane model’s free parameters (including current densities of each current and temperature-dependent effects on the endogenous potassium currents) were fitted to match a representative action potential recorded in a tissue cultured monolayer using a microelectrode, by coupling a multiobjective genetic search algorithm [41–43] with 2-D continuous model simulations. While membrane models have traditionally been constructed in the context of a single isolated cell, several groups have recently attempted to fit a propagating action potential rather than one obtained from an isolated cell in order to account for the electrotonic coupling of neighboring cells [44,45]. Kaur et al. demonstrated that two sets of parameters that generate nearly identical action potentials in an isolated cell model can generate drastically different action potentials in a model of 2-D propagation [42]. In this work, we utilized an approach of fitting both the action potential morphology and the tissue CV simultaneously by searching for the optimal membrane free parameters and the tissue bulk conductivity. Recently, Johnstone et al. showed that fitting with a single action potential was sufficient to accurately estimate up to 6 channel conductances [46], and as such, we believe that our methodology that faithfully reconstructed the Ex293 action potential shape and CV (Fig 2, S1 Table) also allowed us to accurately estimate true channel conductances.

### Introduction of Variation to Replicate Experimental Variability

Variability in ionic currents can lead to changes in steady state macroscopic conduction properties, as well changes in dynamic behaviors such as restitution [47], making its incorporation into computational models critical to accurately predict behavior under arrhythmogenic conditions. Variability in cardiac electrophysiological models has typically been considered on a regional basis (i.e. atria vs ventricles, epicardial vs endocardial [48]); more recent work has recognized the important of modeling other sources of variability including beat-to-beat variation, cell-to-cell variation, and inter-subject variation, as each of these impact measured electrical variability (Fig 8A). Several studies have developed populations of cell models with cell-to-cell variability where model parameters are distributed in a range around the original model parameter values [14,49]. However, the majority of these efforts have focused on modeling and studying isolated single cells, and studies that have developed models of tissues with cell-to-cell variability have generally introduced variability to only a single current [10,50]. Recently, Walmsley et al. conducted a more comprehensive examination of the effects of beat-to-beat variability and cell-to-cell variability in two dimensional tissue simulations and found that while the effects of both are muted in well-coupled tissues, the effect of cell-to-cell variability predominates as tissue coupling is reduced [12]. Finally, it is well-known that electrophysiological properties vary between subjects, and models have been developed to capture this variability by recreating experimental action potentials from different subjects [13,43,51], but no models known to us have used the combination of inter-subject and within-subject variation to explain experimentally recorded variability.

In order to recreate previously reported experimental variability in Ex293 behavior, we sought to identify the type and degree of variation that was required in the Ex293 model [7,9]. We chose to model only cell-to-cell variation and inter-subject variation because the effect of beat-to-beat variation is largely masked by that of cell-to-cell variation [12,52]. Inter-subject (with monolayers being considered as different subjects) variation of ionic currents could result from small differences in culture conditions, as well as from variability in the properties of cells used to initially seed each monolayer. In addition to inter-monolayer ionic conductance variation, we also considered inter-monolayer conductivity variation, which would result from variability in monolayer confluence and degree of coupling at the time of experimental recordings. We found that only combined “triple variation” in cell-cell conductance, inter-monolayer conductance, and inter-monolayer conductivity was necessary and sufficient to replicate experimental variability in single cell properties, and mean monolayer APD and CV (Fig 3), as well as several other properties including resting membrane potential and action potential amplitude (S2 Table, S5 Fig).

The need for multifactorial variation is congruent with analysis of experimental intra-monolayer variability (S2 Fig). Experimental variability in APD within a single monolayer is substantially smaller than the variability between monolayers, indicating that the strong coupling within each monolayer masks any inherent cell-to-cell APD variability. However, the degree of cell-to-cell upstroke velocity variability within a single monolayer is comparable in magnitude to inter-monolayer upstroke velocity variability, indicating that both forms of variation are essential in recreating overall experimental variability. While a model that only considered inter-monolayer variability could feasibly reproduce the range of macroscopically observed conduction behavior under normal conditions, small differences in local upstroke velocity could be crucially vital to determining macroscopic behavior (i.e. whether conduction block occurs) under critical regimes of conduction such as reduced excitability and poor coupling.

While we incorporated variation as a normal distribution around the baseline model parameters, others have previously used uniform variation on the range of [-100%,+100%] and performed a post-hoc screening to identify those models whose action potential properties fall within predefined, experimentally-based inclusion criteria [15]. This approach is useful for studying the relative contributions of each current and the interactions between currents, but the post-hoc screening step makes it challenging to use this approach for the generation of numerous tissue models with randomly generated cell-to-cell variability. Instead, we chose to vary each model parameter around a baseline value determined using a representative action potential recording, as described above, with a degree of variation selected to match the distribution, rather than the range, of output properties. Both this method, and the uniform distribution method with post-hoc selection resulted in a normal-like distribution of output properties (S5 Fig, and Fig 3 of [15]). While our method of normally distributed variation might be less suited for mechanistic or sensitivity analysis, it allows for the simulation of tissues where the properties of each node are randomized and determined at the time of simulation initiation without the need for calibration or screening.

### Model Validation in Diverse Conditions

In order to use computational simulations and derive meaningful conclusions from their results, the underlying model must be validated under conditions beyond a single action potential measured at a regular pacing rate. It has previously been noted that variation in channel conductance can lead to significant variation in restitution behavior [47] and this effect is clearly seen in experimental restitution behavior. The Ex293 membrane model with triple variation was able to closely match both the mean degree and the variability of experimental restitution behavior (Fig 5) with CV restitution being recapitulated more closely than APD restitution. We note that the model CV restitution curve is steeper than that of APD restitution, a behavior that is seen across other experimental and computational studies; however, the Ex293 experimental APD restitution curve is much steeper than the CV restitution curve, suggesting that additional study of this anomalous relationship is warranted.

In addition to modeling restitution properties, we examined how tetrodotoxin (TTX), barium chloride (BaCl_2_), and the extracellular potassium concentration ([K^+^]_o_) affect conduction in simulated Ex293 monolayers compared to experimental observations. The simulated response of the Ex293 model to TTX and BaCl_2_ was qualitatively similar to that in experimental monolayers (Fig 6). Under BaCl_2_ treatment, however, the minimum achieved CV before failure was lower experimentally than in the model, and consequently, the experimental results exhibited longer APD than the model. This is likely because the discrete nature of experimental monolayers leads to local variation in conductivity that allows for propagation of a slow, non-planar wavefront that cannot occur in a continuous representation of tissue. We also note that direct comparison of experimental and simulated results is not possible in the absence of a dose-response relationship; as such differences in the shape of the BaCl_2_ response curves may be due to scaling of figure axes, or to uncertainty in voltage-dependent model of BaCl_2_ block. Furthermore, the Ex293 model and experimental monolayers behave similarly when subject to increased [K^+^]_o_, although the model failed to conduct at lower [K^+^]_o_ than experimental monolayers. This may be because the changes in reversal potential due to changing [K^+^]_o_ are slightly less than predicted by the Nernst formula [32], which would lead to less sodium inactivation and facilitate conduction in cases where the model predicted failure. Such modification of the model resulted in a [K^+^]_o_ failure threshold comparable to that observed experimentally, suggesting that modification of biophysical models to include “real-world” behavior is necessary in order to faithfully simulate the complexity of experimental preparations.

### Impact of Spatial Organization of Variation

The importance of cell-to-cell ionic conductance variation in reproducing experimental variability raises the question of whether ionic properties should be varied randomly across a tissue or whether a more complex spatial organization of variation is needed. Because *in vitro* and *in vivo* tissues develop through the repeated growth and division of an initial population of cells, it is conceivable that each tissue contains regions of reduced variance due to common cellular lineage, paracrine effects, and local metabolic conditions. We thus analyzed an extreme case of spatial organization, where the central region of the tissue exhibits reduced I_K1_ conductance and reduced ionic conductance variance (Fig 8A) and found that the presence of a spatial organization of variation did not significantly impact conduction behavior in well-coupled monolayers (Fig 8B–8D). Because the strong coupling can mask effects of cell-to-cell variation, the impact of functional decoupling of the monolayer through simulated fibrosis was examined. Simulated conduction in fibrotic tissue with random cell-to-cell variation showed conduction slowing at basal pacing rates, a further exaggerated slowing at shorter diastolic intervals, and an increase in variability in the minimum diastolic interval able to sustain conduction across the tissue (Fig 8B–8E), compared to control well-coupled tissue, in line with previous studies [53,54]. The addition of spatial organization of variation to fibrotic tissue had no significant impact on macroscopic conduction at 1 Hz pacing (Fig 8B), or at shortened diastolic intervals (Fig 8C and 8D). In addition, conduction failure behavior in these tissues was similar to fibrotic tissues without a central region (Fig 8E).

These results suggest that cell-to-cell variation can be incorporated randomly into a tissue model without the consideration of the spatial distribution of that variation. In addition, while it is clear that fibrosis increases arrhythmogenic potential by slowing conduction and inducing premature conduction failure, it appears unlikely that the presence of fibrosis unmasks any additional pro-arrhythmogenic effect from the variability of cellular properties of the underlying tissue.

### Conclusions

This study describes a new computational model of the engineered excitable Ex293 cell that reproduces experimentally observed behavior in a range of normal and abnormal conduction conditions. We have identified the key components of experimental variability that are necessary to include in the model–namely, inter-monolayer conductivity variation, and cell-to-cell and intra-monolayer ionic conductance variation—and implemented a simple yet novel method of stochastic normal random variation to allow for the simulation of the full range of experimental outcomes rather than simply the mean. While experimental approaches are limited in their ability to simultaneously gather data at high spatial and temporal resolution, computational simulations can provide tissue-wide high resolution recordings that can help elucidate the subcellular electrophysiological mechanisms behind observed macroscopic behavior. As such, we believe that this paired experimental-computational platform will enable unique future insights into the effects of microstructural variation on microscopic and macroscopic impulse conduction

### Limitations

The computational model of Ex293 cell was developed under the assumption that the tissue is a continuum rather than discrete structure with individual cells and sub-cellular regions. Such a formulation may fail to capture the effects of changes in tissue properties or electrical behavior that occur on the spatial scale of individual cells (for example, [55]). However, the validation studies were performed in well-coupled tissue and most tissue structural changes either occur on larger spatial scales (e.g. collagen deposition) or can be simulated via alteration of local properties in the continuous model (e.g. tissue decoupling, cell death etc). In addition, the Ex293 model can easily be incorporated into a discrete model of tissue structure, as previously described by our group [56], if necessary for further replication of experimental observations.

Our model incorporates only two of the endogenous currents that have been identified in HEK293 cells—the endogenous potassium and sodium currents. Chloride currents were excluded from the model because they are poorly characterized under physiological conditions, and there remains significant uncertainty as to their rectification behavior, calcium dependence, and peak density. In addition, we chose not to include endogenous calcium currents because of their relatively small magnitude compared to the other currents [35]. The voltage dependence of the described endogenous calcium currents appears qualitatively similar to that of the endogenous and transfected sodium currents [37], and given the transfected sodium current conductance in our model is approximately 100x the literature-reported conductance of the endogenous calcium current, exclusion of the calcium current from the model likely had minimal effect, even in cases of partial sodium channel blockade.

While the degree of channel conductance variation is likely different for each channel type, we considered a single degree of variation for all channels because of limited information into each channel’s variability. In addition, our model does not incorporate cell-to-cell variability in tissue conductivity. While the variations used in this work were able to reproduce the variability seen in experimental results, the degrees of inter-monolayer variation in different experimental set will need to be modified to match specific experimental variability due different culture conditions, monolayer seeding and handling.

## Methods

### Model Development

The excitable cell membrane of a single cell is modeled by the following differential equation:

$$C_{m}\frac{dV_{m}}{dt}\;=\;-\left(I_{stim}+I_{ionic}\right)$$ (1)

where *C*_*m*_ is the membrane capacitance, *V*_*m*_ is the transmembrane voltage, *I*_*stim*_ is the externally applied stimulus current, and *I*_*ionic*_ is the sum of the individual ionic currents (whose dynamics are described by a system of ordinary differential equations) that contribute to the action potential:

$$I_{ionic}\;=\;I_{Na}+\;I_{K}+\;I_{Na,wt}+\;I_{K,wt}$$ (2)

#### Transfected inward rectifying potassium current (I_K1_)

The I_K1_ current was modeled with a single activation gate, *n*, with fast and slow components (n_1_ and n_2_, respectively), and a dependence on extracellular potassium concentration:

$$I_{K1}\;=\;G_{K}\;\sqrt{\frac{\left[K_{ext}\right]}{5.4}}\;\left(f*n_{1}+\left(1-f\right)*n_{2}\right)\;\left(V_{m}-\;E_{K}\right)$$ (3)

where f is a constant that describes the relative contribution of the fast activation component, and [K_ext_] is the extracellular concentration of potassium. The steady state values of the activation gate (*n*_∞_) was described as a function of membrane potential by a Boltzmann sigmoid curve, $n_{\infty }\;=\;\left(1-n_{res}\right)/\left(1+\;e^{\left(V-V_{1/2}\right)/k}\right)+n_{res}$, where V is the membrane potential, V_1/2_ is the voltage at which *n*_∞_ = 0.5, k is the slope factor, and *n*_*res*_ is the residual value of the gating parameter, independent of membrane potential. Parameters for the Boltzmann equation were determined by fitting experimental data from Kirkton and Bursac [7], obtained as described therein, corrected by subtracting currents measured in wild-type HEK293 cells using the same protocols. The mean currents were then divided by the driving force (V_test_ − E_K_), where E_K_ is the potassium reversal potential, and normalized before fitting to the Boltzmann curve.

Time constants of activation as well as the relative contribution of the fast and slow activation components were determine by fitting experimental voltage traces following step voltage change from a holding potential of -40 mV. Voltage traces were converted to conductance traces by dividing by the driving force (*V*_*m*_ − *E*_*K*_) and then normalized across all traces. Each resulting conductance trace for test potential *v* was then fit to the equation:

$$g\left(v,t\right)\;=\;n_{\infty }\left(v\right)\;\left(f*\left(1-e^{-\frac{t}{\tau_{n1}\left(v\right)}}\right)+\left(1-f\right)\left(1-e^{-\frac{t}{\tau_{n2}\left(v\right)}}\right)\right)$$ (4)

where, *n*_∞_ is the previously determined value of the activation gate at the test potential, *τ*_*n1*_ and *τ*_*n2*_ are the unknown fast and slow activation time constants, respectively. Fitted values of *τ*_*n1*_ and *τ*_*n2*_ were then used to formulate a functional definition of the time constants as a function of membrane potential.

A Q10 value of 1.419 was used to adjust model time constants to account for dynamic differences between behavior at 23°C and 35°C, based on temperature dependence studies in guinea pigs from Martin et al. [22]. In addition, a 8.25 mV left shift in the V_1/2_ of the potassium steady state activation curves was determined by using data from Martin and by assuming linearity in temperature dependence, as previously done by others [57].

#### Transfected fast voltage-gated sodium current (I_Na_)

The I_Na_ current was modeled with three identical activation gates, and one inactivation gate with fast and slow components, similar to the work of Lindbald et al. [58]:

$$I_{Na}\;=\;G_{Na}m^{3}\;\left(dh_{1}+\left(1-d\right)h_{2}\right)\;\left(V_{m}-\;E_{Na}\right)$$ (5)

where m is the sodium activation gate, *h*_1_ and *h*_2_ are the fast and slow components of the inactivation gate, respectively, and d is a constant that describes the relative contribution of the faster inactivation component. While both *h*_1_ and *h*_2_ have the same steady-state value (*h*_∞_), they differ in their rate of inactivation, *τ*_*h*1_ and *τ*_*h*2_. The steady state inactivation curve (*h*_∞_) was described by a Boltzmann sigmoid curve, fit to data from Kirkton and Bursac [7], obtained by applying a 20 ms test pulse at -20 mV after a 500 ms inactivating pre-pulse at voltages ranging from -130 mV to -40 mV. As explained in the *Supplementary Text 1*, the steady state activation curve, as well as activation and inactivation time constants were determined by fitting of experimental sodium current recordings following depolarization from a holding potential of -100 mV. The recordings were converted to conductance traces and normalized to the overall maximum conductance across all test potentials; the resulting traces were then each fitted to the equation

$$g\left(v,t\right)\;=\;m_{\infty }{\left(v\right)}^{3}\;e^{-\frac{t}{\tau_{m}\left(v\right)}}*h_{\infty }\left(v\right)*\left(d*\left(1-e^{-\frac{t}{\tau_{h1}\left(v\right)}}\right)+\left(1-d\right)\left(1-e^{-\frac{t}{\tau_{h2}\left(v\right)}}\right)\right)$$ (6)

where *h*_∞_ is the value of the previously defined sodium inactivation gate, and *m*_∞_ was allowed to be a fitted parameter along with the time constants of activation (*τ*_*m*_) and inactivation (*τ*_*h1*_ and *τ*_*h2*_) as well as the relative contribution of the two inactivation components (d). The resulting steady state activation curve was normalized across voltages and based on its shape, fitted to the form of the sum of two Boltzmann sigmoid curves. The inactivation time constants measured for test potentials above -45 mV were combined with time constants from recovery from inactivation to define the functional definitions of the inactivation time constant.

Time constants were temperature-adjusted using a Q_10_ = 2.79, as calculated by Ten Tusscher et al. [57] using data from Nagatoma et al. [21]. Rightward shifts of 5.16 mV and 5.64 mV in the V_1/2_ of the sodium steady state activation and inactivation curves, respectively, were determined using data from Nagatoma and by assuming linearity in temperature dependence.

#### Endogenous sodium current (I_Na,wt_)

The I_Na,wt_ current was modeled based on the Hodgkin and Huxley formulation with three identical activation gates and one inactivation gate.

$$I_{Na,wt}\;=\;G_{Na,wt}o^{3}p\;\left(V_{m}-\;E_{Na}\right)$$ (7)

Because of limited experimental data on the endogenous channel in Kirkton and Bursac [7], the steady-state activation and inactivation curves as well as the relevant time constants for the endogenous sodium current were derived from the findings of He and Soderlund [16]. The inactivation curves was right-shifted by 5.76 mV to account for difference in temperature, and time constants were scaled with a Q_10_ of approximately 3 based on temperature dependence data of Na_v_1.7 channels from Han et al. [59],

#### Endogenous potassium current (I_K, wt_)

The I_K, wt_ current was modeled using the form of a slow delayed rectifier current from Ten Tusscher et al. [57], with two identical activation gates:

$$I_{K,wt}\;=\;G_{K,wt}b^{2}\;\left(V_{m}-\;E_{K}\right)$$ (8)

The steady state activation curve and activation time constant were determined by fitting to activation data at 22°C from Yu and Kerchner [17]. Because the molecular identity of the endogenous potassium current is unknown [19], temperature-dependent changes in the activation curve and the time constant were used as free parameters during the fitting process.

### Model Fitting

A multi-objective genetic search algorithm was used to identify the optimal ion channel conductances, tissue conductivity and other free parameters necessary to reproduce the experimental action potential waveform and CVs in Ex293 monolayers. An initial population of 600 “parent” parameter sets was generated by randomly choosing values from the physiologically reasonable search range for each parameter (Table 1). A custom MATLAB script ran the 2-D tissue simulation for each trial parameter set (see *Numerical Methods*), and computed the values of the two error functions: the root mean squared error (RMSE) between an experimental AP recording and the simulated AP, and the absolute difference between the experimental and simulated CV. 2-D simulation was performed in a 140 node x 140 node monolayer (dx = dy = 50 micron) with no-flux boundary conditions. The tissue domain was paced at 1Hz at one corner and the action potential tracing of the third action potential from a node 0.6 cm diagonally from the stimulus site was recorded. CV was determined from activation times (50% AP amplitude) at nodes 0.2 cm and 0.8 cm from the stimulus site. RMSE was calculated by aligning the experimental action potential (recorded via sharp electrode in [7]) and simulated action potentials at the upstroke crossing of -40 mV.

The multi-objective genetic algorithm was configured to run in parallel across 32 cores using MATLAB’s Parallel Computing Toolbox and Global Optimization Toolbox [60], with heuristic crossover, tournament selection and a small Pareto fraction, and terminated when the average change in the spread of the Pareto front was less than 0.001 over 50 generations. The genetic algorithm was run 9 times to examine diversity of results; the parameter set that minimized the RMSE was selected from each Pareto front.

### Data Analysis

In order to compare macroscopic conduction properties of simulation results with those obtained experimentally using a voltage sensitive dye and an optical fiber recording array, averaging of local simulated membrane potentials in small circular regions was performed to simulate optical recording of model results. While the experimental optical fiber array has fibers with diameter of 750 μm arranged in a 20-mm diameter hexagonal bundle, spacing between the optical fiber array and the tissue sample results in a wider effective field of view for each fiber. As a result, while our simulated optical sensors were spaced with 750 μm center-to-center spacing, each sensor averaged potentials over a circular region of diameter 1100 μm. The resulting voltage traces for each of the 504 simulated optical sensors was analyzed using custom MATLAB software developed for analysis of experimental optical mapping recordings, and the CV and APD_80_ (action potential duration at 80% repolarization) were calculated, as previously described [5,7].

### Incorporation of Variation and Sensitivity Analysis

Inter-monolayer variation of conductances and conductivity was generated by selecting scaling factors for each monolayer from random normal distributions with mean 1 and specified standard deviation. Base model parameters were then scaled by these factors and the new monolayer-specific mean parameters were provided to the Cardiowave simulator. Cell-to-cell variability was incorporated directly into the membrane model. Each node’s channel conductance is randomly selected at the start of the simulation from a random normal distribution with the selected mean monolayer channel conductance and a specified standard deviation (see Fig 4B). Simulated variability was compared to experimentally variability measured in [7] including macroscopic CV recorded via optical mapping in n = 39 independent monolayers, and single cell APD and maximum upstroke velocity recorded via sharp electrode recording in 6 different (biological replicate) monolayers (n = 4–5 cells per monolayer; 27 total recordings).

The sensitivity of the membrane model to perturbation was assessed by independently altering the conductance of each current in a range from 50% to 150%, and measuring the impact on conduction properties. Sensitivity was measured in a simulated strand of tissue (600 x 10 nodes; dx = dy = 20 micron, no-flux boundary conditions). In addition to the macroscopic conduction properties measured using simulated optical sensors, several addition properties were measured using the action potential traces from the center-point of the strand, analogous to experimental sharp electrode recording. These properties included the maximum upstroke velocity (dV_m_ dt^-1^), resting membrane potential, APD and action potential amplitude.

### Validation Studies Using the Membrane Model

Restitution of APD and CV was measured in a 2D strand continuous monodomain model (600 x 10 nodes; dx = dy = 20 micron). The standard (S1-S2) protocol was used wherein the strand was stimulated from one end at 2 Hz for 10 pulses (S1) followed by a premature stimulus of the same amplitude (S2), and the CV and APD resulting from the S2 stimulus were recorded. The S1-S2 interval was decreased until the S2 pulse no longer elicited an action potential.

Pharmacological channel blockade due to tetrodotoxin (TTX) was simulated by simultaneously altering the conductance of both the I_Na_ and I_Na,wt_ currents. Blockade of the I_K1_ channel due to BaCl_2_ was simulated by scaling the I_K1_ conductance based on the degree of block at -100 mV and the membrane potential (S4 Fig). The effect of varied extracellular potassium concentration was simulated by scaling the sodium conductance proportional to the square root of extracellular potassium, as included above in eq 3, and by altering the reversal potential of potassium, as predicted by the Nernst equation. The physiological intracellular potassium concentration was estimated by assuming that the resting potential during sharp electrode recordings of HEK cells transfected with only the Kir2.1 channel (with control extracellular potassium concentration of 5.4 mM) is equivalent to the potassium Nernst potential [7]. The reversal potential as a function of extracellular potassium is then calculated using this estimate of intracellular potassium concentration.

### Impact of Spatial Organization of Variation

Conduction was simulated in a 2D continuous monodomain model (600 x 200 nodes; dx = dy = 10 micron). Non-conductive fibrosis-like obstacles were added to the tissue domain, with each obstacle 100 μm x 100 μm in size and with 100 μm spacing between obstacles. Obstacles were decoupled from neighboring tissue nodes to establish no-flux boundary conditions. A central region of homogeneity with a diameter of 140 μm (70% of strand width) was established. Within this region, the mean potassium conductance was one standard deviation (12.5%) below the monolayer mean, and the standard deviation of cell-to-cell variation was narrowed to 0.0625 for all ionic properties to create relative homogeneity with preserved but reduced cell-to-cell variability. Cell-to-cell ionic variation in the remainder of the tissue was resampled in order to preserve the distribution of cell-to-cell variation across the full tissue. The central region remained fully coupled to the surrounding tissue. The previously described standard S1-S2 protocol was used to assess restitution behavior.

### Numerical Methods

All simulations were performed using the Cardiowave software package [20], a cardiac simulation system that incorporates numerous modules for various membrane models, time integration methods and linear solvers (available online at cardiowave.duke.edu). The governing equations were discretized using finite differences and propagation was simulated using a semi-implicit Crank-Nicholson scheme with adaptive time steps between 5 μs and 100 μs. A biconjugate gradient stabilized method solver with tridiagonal preconditioner was used to simulate each time-step. Potentials were recorded at intervals of 10 μs at selected individual points and across the domain using spatial averaging across simulated optical sensors, as described earlier.

### Statistical Methods

All data is presented as mean +/- standard deviation unless otherwise specified. Comparison of model and experimental variability was performed using Levene’s test for equality of variances with an alpha value of 0.05. Comparison of macroscopic CV, APD and failure behavior in the presence of fibrosis and spatial organization of variability was performed using a two-way ANOVA with two between-subjects measures (fibrosis and organization). Comparison of restitution behavior was performed using three-way ANOVA with one within-subjects measure (S1-S2 interval) and two between-subjects measures (fibrosis and organization), and post-hoc pairwise comparison was performed using Fisher’s LSD.

## Supporting Information

S1 Text Supplementary discussion of methodology of ion channel modelling. (PDF)

S1 Fig Comparison of membrane currents. (A) Experimentally recorded currents during “AP-clamp”. Note that the duration of the experimental action potential used to generate this recording is longer than the mean duration which was matched by the model. (B) Action potential trace used for simulated AP clamp (C) Model currents at 23C qualitatively matches experimental currents. (D,E) Model currents at 35C. Temperature induced variation in conductances and activation/inactivation properties lead to a much larger inward sodium current.
(PDF)

S2 Fig Intra-monolayer variability. Standard deviations of measured single cell APD and upstroke velocity were obtained for each experimental monolayer (n = 6). Monolayers exhibit relatively little APD variability but substantially more maximal upstroke velocity variability within each monolayer. The standard deviation of each parameter between monolayers is indicated by the dashed red line. The degree of cell-to-cell upstroke velocity variation within individual monolayers is comparable to the variation seen between different monolayers, while cell-to-cell variation of action potential duration within individual monolayers is 3-5x smaller than that seen between different monolayers. Models that include only inter-monolayer variability and not cell-to-cell variability fail to reproduce the experimentally observed variability of upstroke velocity within individual monolayers.
(PDF)

S3 Fig Sensitivity analysis. Current densities of each of the four constitutive currents were independently varied from 50% to 150% and the effect of on conduction and action potential shape properties was measured. Variation in I_Na_ and I_K_ led to changes in both CV and APD while variation of the endogenous currents affected APD without affecting CV. Variation in both I_Na_ and I_K,wt_ led to changes in action potential amplitude, while only I_Na_ variation affected the maximal upstroke velocity.
(PDF)

S4 Fig Model of voltage and dose dependent barium chloride induced block. IK1 block due to barium chloride was modeled as dependent on membrane potential and degree of block at -100 was used as a substitute for drug dose.
(PDF)

S5 Fig Simulated variability in single cell properties. The model is able to replicate experimental variability in several isolated single cell properties other than those used to set levels of conductance variation.
(PDF)

S1 Table Comparison of model and experimental Ex293 cells. (PDF)

S2 Table Model equations. (PDF)
