# Supplementary material for: Modeling an Excitable Biosynthetic Tissue with Inherent Variability for Paired Computational-Experimental Studies
Source: PLoS Comput Biol. 2017 Jan 20;13(1):e1005342. doi: 10.1371/journal.pcbi.1005342 (PMC5291544; doi:10.1371/journal.pcbi.1005342)
Supplement: S1 Text — (PDF) [file pcbi.1005342.s001.pdf]

Supplementary Text 1.

The two transfected currents and two endogenous currents were modeled using Hodgkin-Huxley type formulations. While more complex Markovian models are convenient for describing specific states and state-transitions of each ion channel [1], insufficient experimental data can lead to non-unique models with unidentifiable parameters that increase computational complexity without significant gain in understanding. Consequently, it is preferable to use Hodgkin-Huxley type models in cases of limited experimental data [2]. Model currents were constructed to simulate behavior at near-physiological temperature (35°C), for direct comparison with 2-D and 3-D cell culture studies. Because most patch clamping is performed at room temperature, the temperature dependence of channel properties had to be considered; in some cases, sufficient literature data was available to adjust for the effect of temperature, while in others, literature data was lacking, and the temperature dependence was left as a free parameter for the fitting process

The I_{K1} current, carried by the Kir2.1 channel, controls the rapid stage of repolarization and has traditionally been described as a time-independent background current [3], because of its rapid activation dynamics. However, given the relatively short action potential duration of the Ex293 cell, and in order to match the activation dynamics observe experimentally, the I_{K1} current was modeled as time-dependent, with a single activation gate. In describing the time-course of activation, we found that the activation kinetics at low voltages could not be adequately described by a single time constant; instead our model uses a fast time constant responsible for 75% of activation, and a slow time constant responsible for the remaining 25% of activation. While the two time constants are equivalent at high voltages (> 0 mV), they differ by up to an order of magnitude at some lower voltages (-100 mV to -80 mV). We were unable to find any previous studies describing the temperature dependence of human I_{K1} currents; however, Martin et al [4] previously studied the effects of temperature on I*K1* currents in guinea pig and cat ventricular myocytes. Because several previous models have used guinea pig data to adjust human currents, we chose to use this data to temperature-adjust our model.

The transfected $Na_v1.5$ protein codes for the alpha subunit of the voltage gated sodium channel which carries the I_{Na} current. This fast voltage-gated sodium current is responsible for the action potential upstroke in cardiac cells, and it plays the same role in the Ex293 cell. The I_{Na} current was modeled based on previous work of Lindblad et al [5], with three identical activation gating particles (m^3) and one inactivation gate with a fast and a slow component. This form of the inactivation gate is likely a simplified but effective approximation of a more complex inactivation gating process that involves multiple closed states. Based on fitting to experimental traces, the fast component was determined to be responsible for 90% of inactivation while the slow component was responsible for the remaining 10%. The slow component of inactivation is approximately 6x slower than the fast component at high potentials, while at low potentials (< -70mV), the slow component is 20x slower. These results are in-line with previous studies of native cardiomyocytes ([6], [7]), and expression of the sodium channel alpha submit in HEK293 cells [8], [9]. While slow inactivation plays a relatively minor overall role, its inclusion in our model is crucial in order to recapitulate the dynamic restitution properties observed experimentally.

Time constants for sodium activation and inactivation, as well as the steady state activation curve, were obtained from fitting current traces elicited by depolarizing pulses. It was noted that the fast inactivation of the sodium current in Ex293 cells occurs at same time scale as the activation of the sodium current. The conventional method for determining the steady state activation curve of the sodium current - a comparison of the maximal currents elicited by stepped depolarization under patch clamp - relies on the assumption that even fast inactivation occurs an order of magnitude or more slower than activation. If this assumption holds, the peak current measured following depolarization to a specific test potential is affected only by the degree of activation at this test potential. However, in the Ex293 cells, the rapid rate of inactivation would mean that the peak current measured following a depolarizing step is affected not only by current activation but also by the onset of inactivation. While the rate of fast inactivation is two orders of magnitude slower than the rate of activation at low voltages (below resting voltage), in the range from -45 mV to 20 mV rates of fast inactivation are less than an order of magnitude slower than rates of activation. The difference is smallest at approximately -25 mV where the activation occurs only twice as fast as

inactivation, meaning that the conventional method for determining steady state activation would be grossly inaccurate in this range of potentials. Similar ratios of activation and inactivation time constant have been observed by others when Nav1.5 channels were expressed in HEK293 cells [10]. The method of fitting current traces to determine activation and inactivation properties, which was used by Hodgkin and Huxley [11], allows for the accurate estimation of steady state activation despite the unusually fast inactivation seen in Ex293 cells. The steady state activation values obtained by current trace fitting were described by the sum of two Boltzmann functions, and the resulting model allows for reproduction of the sodium I-V curve (Figure 1A, dotted line), which is not possible in a model using the conventionally-determined steady state activation function.

The HEK293's endogenous potassium current exhibits the properties of a slow delayed rectifier current, with activation at high potentials and minimal inactivation, so this endogenous current was modeled using the mathematical description of the I_{Ks} current from Ten Tusscher et al. [12]. The steady state activation curve for the endogenous potassium channel, as well as the time constant of activation were determined based on data from Yu and Kerchner [13]. Several groups have studied the HEK293 endogenous potassium current, but none have reported on its temperature dependence. Further, Jiang et al. [14] found that HEK293 cells expressed genes from several voltage gated potassium channels and therefore, the endogenous potassium current did not have a single molecular identity, making it difficult to extrapolate temperature dependence from known channel data. As a result, the shift in the $V_{1/2}$ and slope factor of the steady state activation curve as well as the time constant of activation were allowed to be free parameters in the later fitting process. By doing so, the fitting routine is allowed to identify the values for these parameters to replicate the action potential recorded experimentally at 35° C. Finally, the steady state activation and inactivation curves for the endogenous sodium current were modeled based on data from He and Soderlund [15]. In addition, the time constants of activation and inactivation were also estimated based on data presented therein. He and Soderlund showed that Nav1.7 channels were responsible for carrying a substantial portion of the endogenous sodium current. Based on these findings, the effect of temperature on

the endogenous sodium current was incorporated into the model using temperature dependence data in Nav1.7 channels from Han et al. [16]

[1] Y. Rudy and J. R. Silva, "Computational biology in the study of cardiac ion channels and cell electrophysiology.," *Q. Rev. Biophys.*, vol. 39, no. July, pp. 57–116, 2006.

[2] M. Fink and D. Noble, "Markov models for ion channels: versatility versus identifiability and speed.," *Philos. Trans. A. Math. Phys. Eng. Sci.*, vol. 367, no. 1896, pp. 2161–79, Jun. 2009.

[3] H. Hibino, A. Inanobe, K. Furutani, S. Murakami, and I. a N. Findlay, "Inwardly Rectifying Potassium Channels : Their Structure, Function, and Physiological Roles," *Physiol. Rev.*, vol. 90, pp. 291–366, 2010.

[4] R. MARTIN, S. KOUMI, and R. TENEICK, "Comparison of the effects of internal [Mg2+] on IK1 in cat and guinea-pig cardiac ventricular myocytes," *J. Mol. Cell. Cardiol.*, vol. 27, no. 1, pp. 673–691, Jan. 1995.

[5] D. S. Lindblad, C. R. Murphey, J. W. Clark, and W. R. Giles, "A model of the action potential and underlying membrane currents in a rabbit atrial cell.," *Am. J. Physiol.*, vol. 271, no. 4 Pt 2, pp. H1666-96, Oct. 1996.

[6] D. J. Wendt, C. F. Starmer, and a O. Grant, "Na channel kinetics remain stable during perforated-patch recordings.," *Am. J. Physiol.*, vol. 263, no. 6 Pt 1, pp. C1234-40, 1992.

[7] Y. Sakakibara, J. a Wasserstrom, T. Furukawa, H. Jia, C. E. Arentzen, R. S. Hartz, and D. H. Singer, "Characterization of the sodium current in single human atrial myocytes.," *Circ. Res.*, vol. 71, no. 3, pp. 535–546, 1992.

[8] O. Moran, M. Nizzari, and F. Conti, "Endogenous expression of the beta1A sodium channel subunit in HEK-293 cells.," *FEBS Lett.*, vol. 473, no. 2, pp. 132–4, May 2000.

[9] P. Tammaro, F. Conti, and O. Moran, "Modulation of sodium current in mammalian cells by an epilepsy-correlated beta 1-subunit mutation.," *Biochem. Biophys. Res. Commun.*, vol. 291, pp. 1095–1101, 2002.

[10] Z. Zhang, Z. Zhao, Y. Liu, W. Wang, Y. Wu, and J. Ding, "Kinetic model of Nav1.5 channel provides a subtle insight into slow inactivation associated excitability in cardiac cells.," *PLoS One*, vol. 8, no. 5, p. e64286, Jan. 2013.

[11] A. L. Hodgkin and A. F. Huxley, "A quantitative description of membrane current and its application to conduction and excitation in nerve.," *J. Physiol.*, vol. 117, no. 4, pp. 500–44, Aug. 1952.

[12] K. H. W. J. ten Tusscher, D. Noble, P. J. Noble, and A. V Panfilov, "A model for human ventricular tissue.," *Am. J. Physiol. Heart Circ. Physiol.*, vol. 286, no. 4, pp. H1573-89, Apr. 2004.

[13] S. P. Yu and G. A. Kerchner, "Endogenous voltage-gated potassium channels in human embryonic kidney (HEK293) cells.," *J. Neurosci. Res.*, vol. 52, no. 5, pp. 612–7, Jun. 1998.

[14] B. Jiang, X. Sun, K. Cao, and R. Wang, "Endogenous K V channels in human embryonic," pp. 69–79, 2002.

[15] B. He and D. M. Soderlund, "Human embryonic kidney (HEK293) cells express endogenous voltage-gated sodium currents and Na v 1.7 sodium channels.," *Neurosci. Lett.*, vol. 469, no. 2, pp. 268–72, Jan. 2010.

[16] C. Han, A. Lampert, A. M. Rush, S. D. Dib-Hajj, X. Wang, Y. Yang, and S. G. Waxman, "Temperature dependence of erythromelalgia mutation L858F in sodium channel Nav1.7.," *Mol. Pain*, vol. 3, p. 3, Jan. 2007.
