# Supplementary figures and images for: Modeling an Excitable Biosynthetic Tissue with Inherent Variability for Paired Computational-Experimental Studies
Source: PLoS Comput Biol. 2017 Jan 20;13(1):e1005342. doi: 10.1371/journal.pcbi.1005342 (PMC5291544; doi:10.1371/journal.pcbi.1005342)

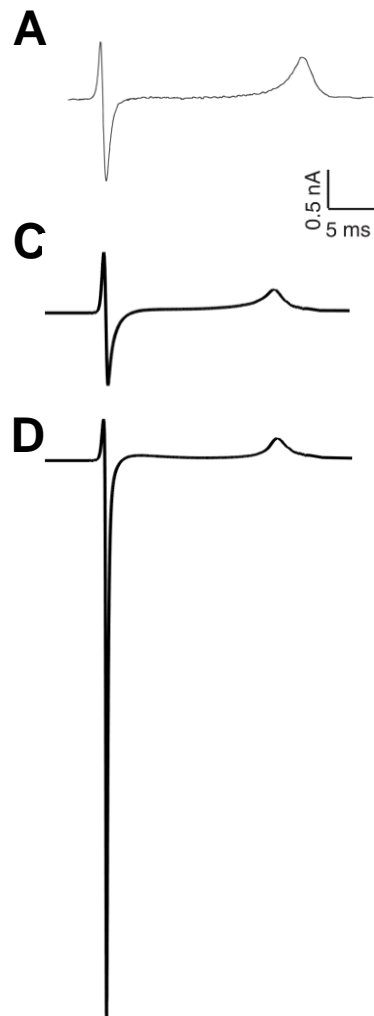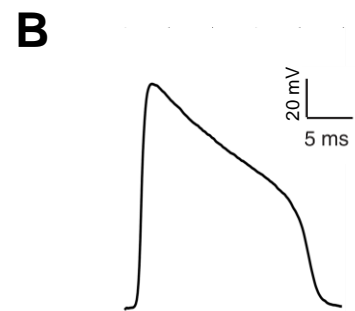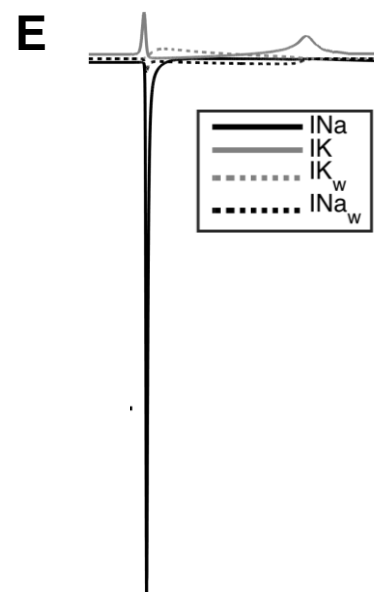

Supplement: S1 Fig — (A) Experimentally recorded currents during “AP-clamp”. Note that the duration of the experimental action potential used to generate this recording is longer than the mean duration which was matched by the model. (B) Action potential trace used for simulated AP clamp (C) Model currents at 23C qualitatively matches experimental currents. (D,E) Model currents at 35C. Temperature induced variation in conductances and activation/inactivation properties lead to a much larger inward sodium current. (PDF) [file pcbi.1005342.s002.pdf]

Standard Deviations of Single Cell APD

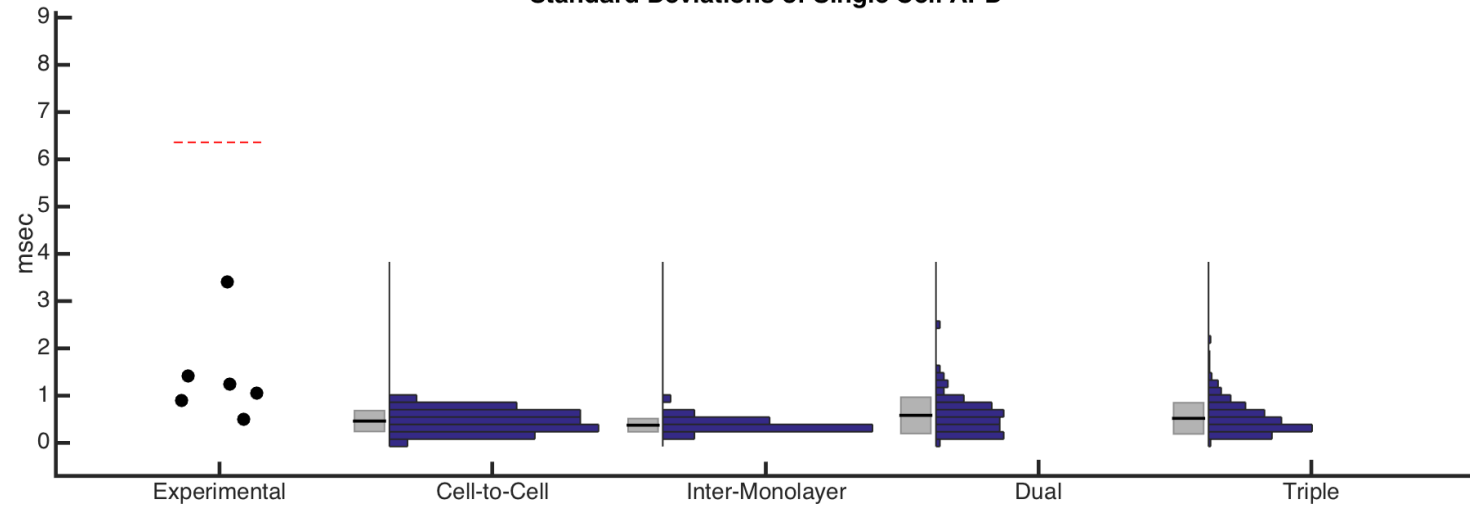

Standard Deviations of Single Cell  $dV/dt_{\max}$

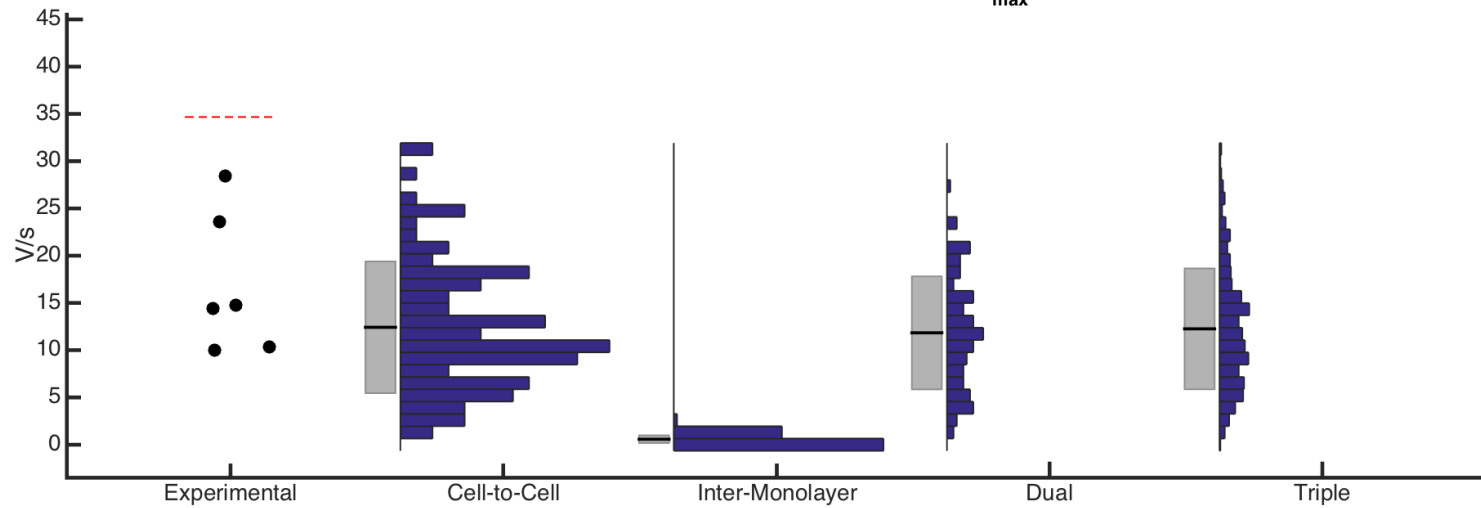

Supplement: S2 Fig — Standard deviations of measured single cell APD and upstroke velocity were obtained for each experimental monolayer (n = 6). Monolayers exhibit relatively little APD variability but substantially more maximal upstroke velocity variability within each monolayer. The standard deviation of each parameter between monolayers is indicated by the dashed red line. The degree of cell-to-cell upstroke velocity variation within individual monolayers is comparable to the variation seen between different monolayers, while cell-to-cell variation of action potential duration within individual monolayers is 3-5x smaller than that seen between different monolayers. Models that include only inter-monolayer variability and not cell-to-cell variability fail to reproduce the experimentally observed variability of upstroke velocity within individual monolayers. (PDF) [file pcbi.1005342.s003.pdf]

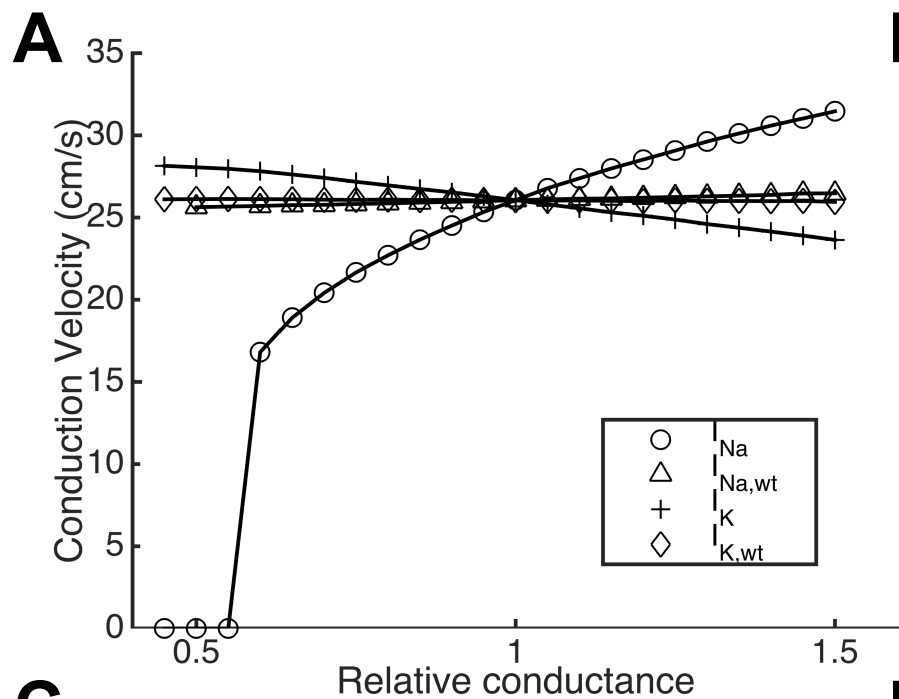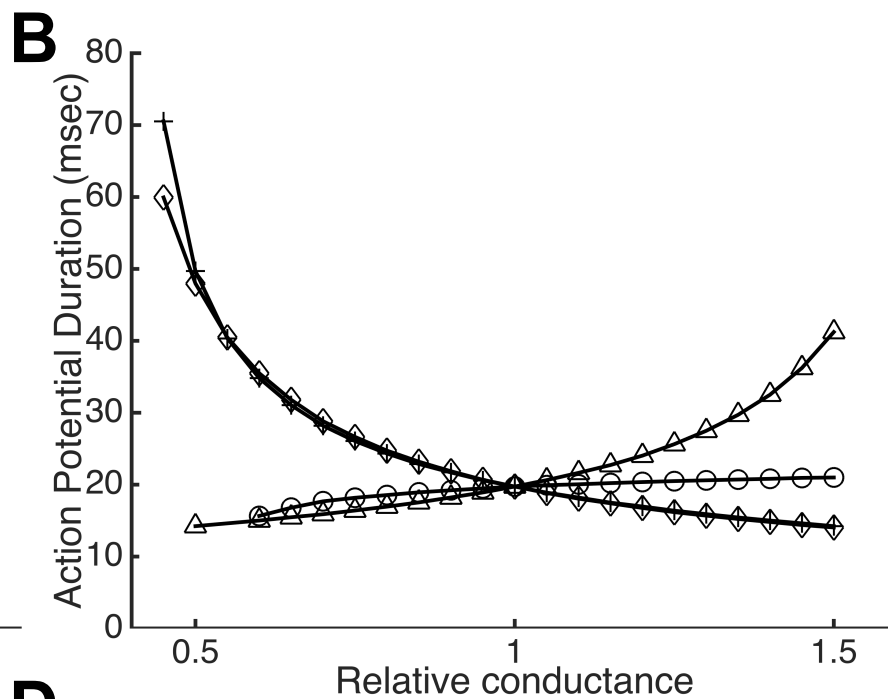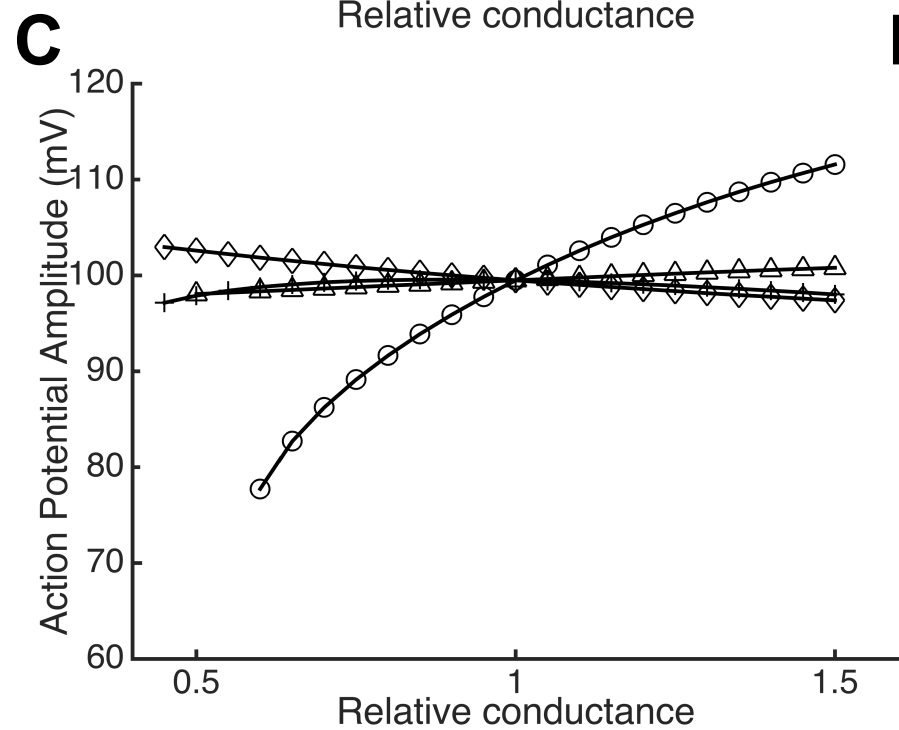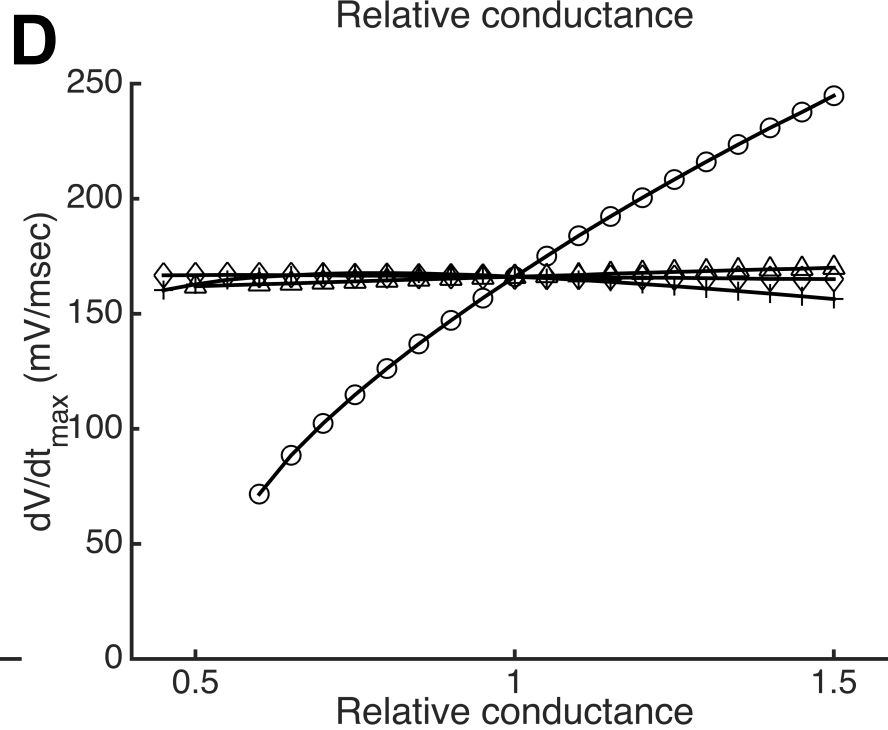

Supplement: S3 Fig — Current densities of each of the four constitutive currents were independently varied from 50% to 150% and the effect of on conduction and action potential shape properties was measured. Variation in INa and IK led to changes in both CV and APD while variation of the endogenous currents affected APD without affecting CV. Variation in both INa and IK,wt led to changes in action potential amplitude, while only INa variation affected the maximal upstroke velocity. (PDF) [file pcbi.1005342.s004.pdf]

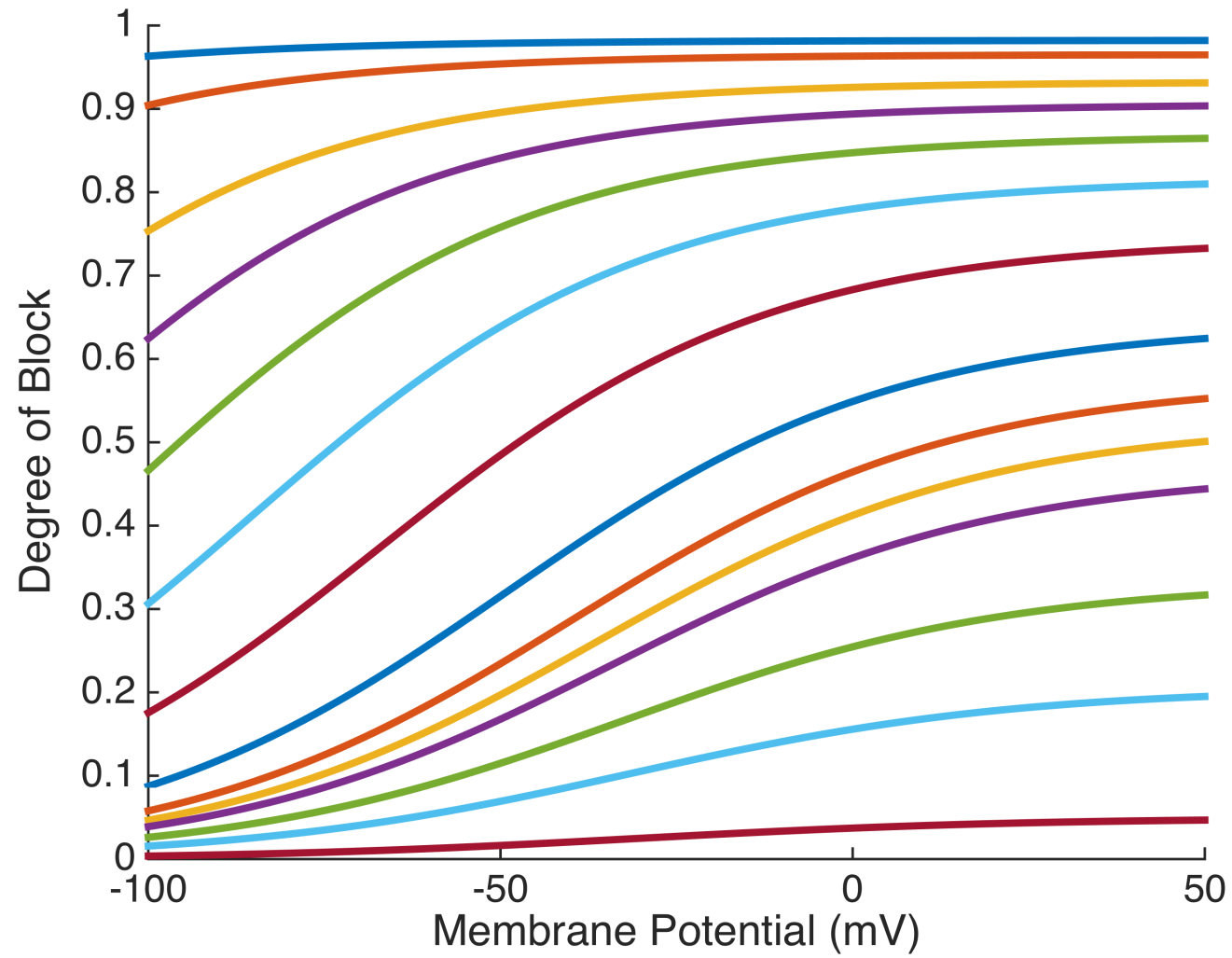

Supplement: S4 Fig — IK1 block due to barium chloride was modeled as dependent on membrane potential and degree of block at -100 was used as a substitute for drug dose. (PDF) [file pcbi.1005342.s005.pdf]

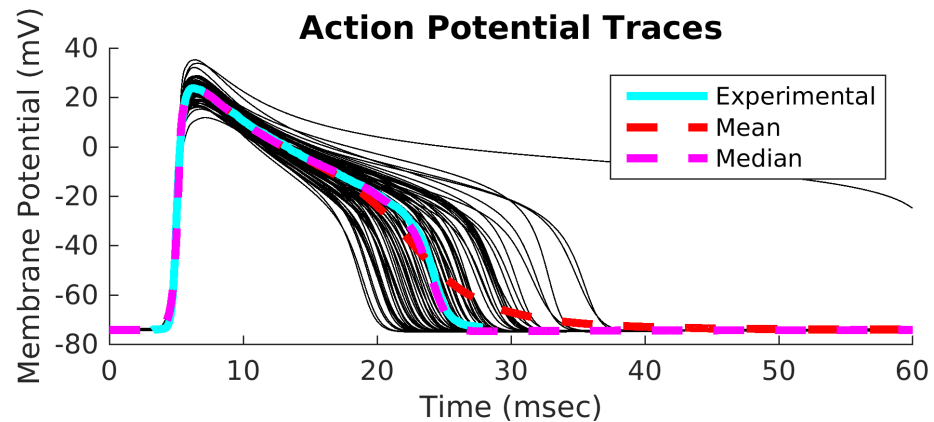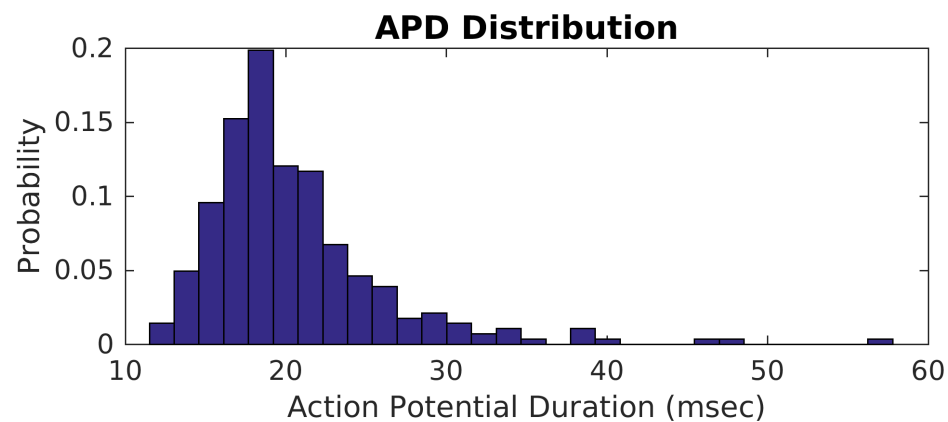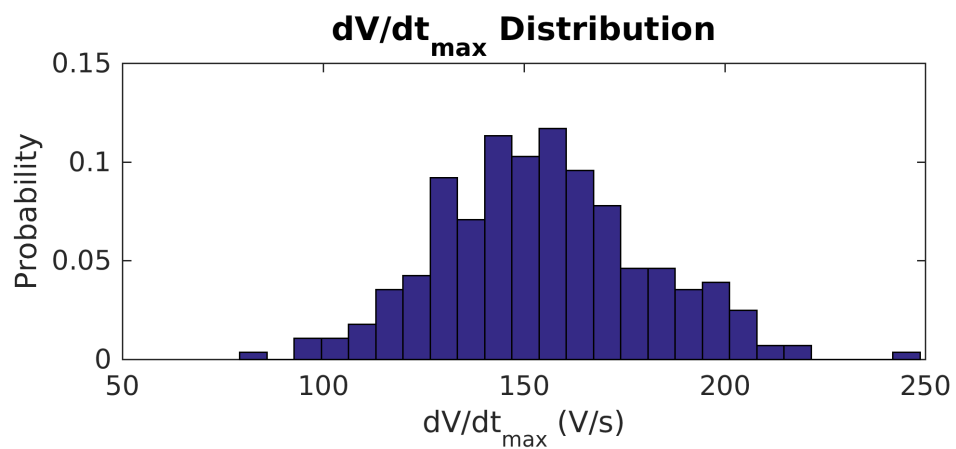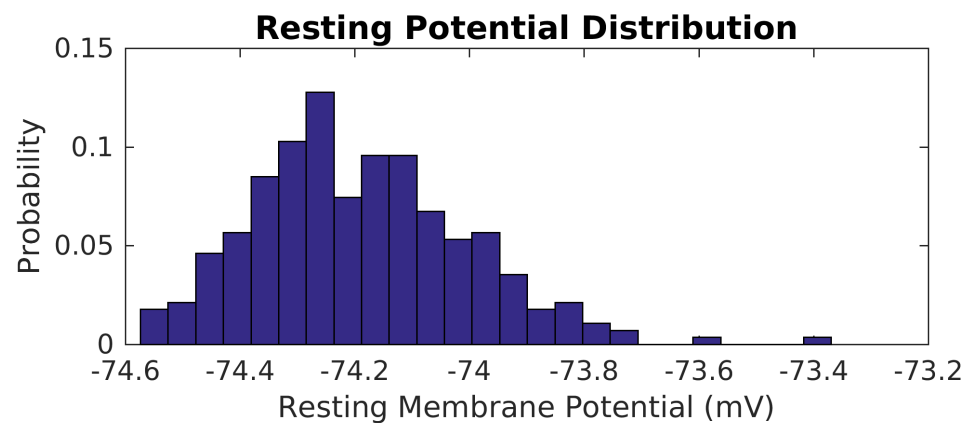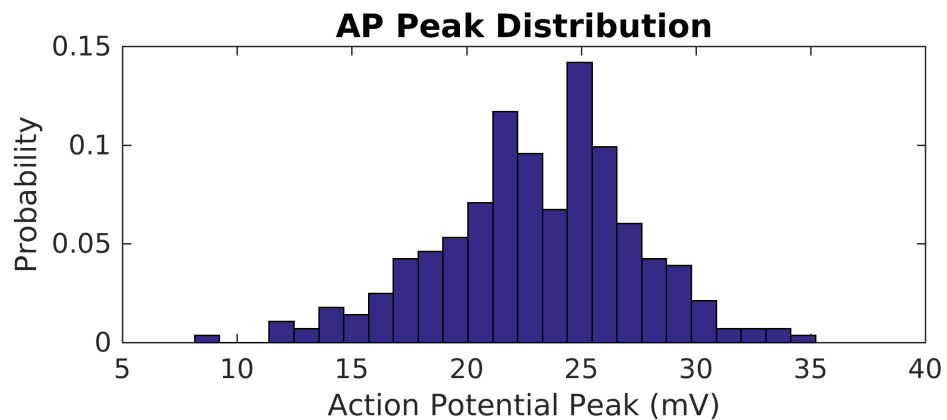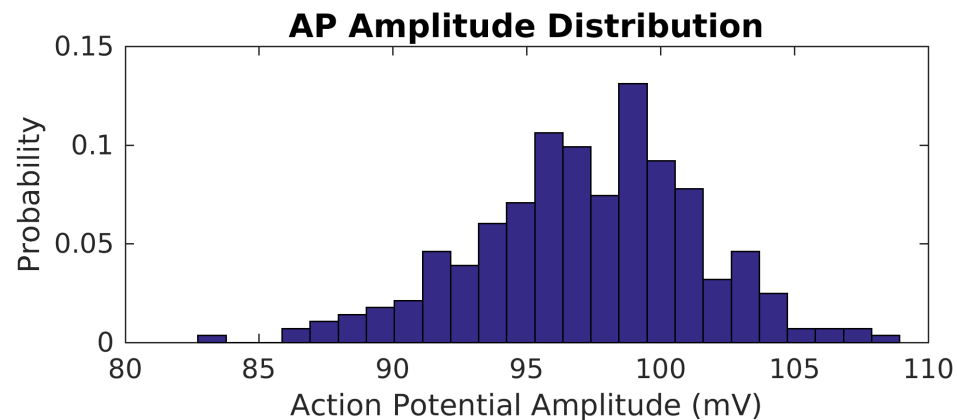

Supplement: S5 Fig — The model is able to replicate experimental variability in several isolated single cell properties other than those used to set levels of conductance variation. (PDF) [file pcbi.1005342.s006.pdf]
