# Supplementary material for: Modeling an Excitable Biosynthetic Tissue with Inherent Variability for Paired Computational-Experimental Studies
Source: PLoS Comput Biol. 2017 Jan 20;13(1):e1005342. doi: 10.1371/journal.pcbi.1005342 (PMC5291544; doi:10.1371/journal.pcbi.1005342)
Supplement: S1 Table — (PDF) [file pcbi.1005342.s007.pdf]

Table S1. Comparison of model and experimental Ex293 cells

	Property	Experimental Mean (SD)	Base Model Value	Variable Model Mean (SD)
Single Cell	Resting potential (mV)	-74.2 (3.64)	-74.2	-74.2 (0.19)
	AP amplitude (mV)	93.3 (5.72)	97.6	97.3 (4.19)
	APD_{80} (msec)	20.9 (5.72)	19.74	21.0 (5.78)
	$(dV_m/dt)_{max}$ (V/s)	150.6 (31.7)	166.07	154.67 (25.8)
	AP peak (mV)	19.0 (5.20)	22.43	23.0 (4.33)
	Conduction velocity (cm/s)	23.3 (3.75)	23.3	23.5 (3.62)
