# Supplementary material for: Modeling an Excitable Biosynthetic Tissue with Inherent Variability for Paired Computational-Experimental Studies
Source: PLoS Comput Biol. 2017 Jan 20;13(1):e1005342. doi: 10.1371/journal.pcbi.1005342 (PMC5291544; doi:10.1371/journal.pcbi.1005342)
Supplement: S2 Table — (PDF) [file pcbi.1005342.s008.pdf]

Table S2. Model equations

Property	Model @ 23C	Model @ 35C
E_{Na}	59.2 mV	61.6 mV
E_K	-72.4 mV	-75.3 mV
G_{Na}	32.64 mS/cm^2	90.34 mS/cm^2
m_{ss}	$\frac{1.653}{1+e^{(V+36.54)/-7.984}} - \frac{0.9225}{1+e^{(V+25.31)/-11.44}} + 0.0971$	$\frac{1.653}{1+e^{(V+31.38)/-7.984}} - \frac{0.9225}{1+e^{(V+20.15)/-11.44}} + 0.0971$
τ_m	$0.9685\, e^{-(V+53.87)^2/1136} + 0.0924$	$0.2827\, e^{-(V+53.87)^2/1136} + 0.0270$
h_{ss}	$\frac{1}{1+e^{(V+86.06)/5.691}}$	$\frac{1}{1+e^{(V+80.42)/5.811}}$
τ_{h1}	$29.40\, e^{-(V+83.17)^2/786.6} + \frac{0.0922}{1+e^{(V-9.195)/2.982}} + 0.6461$	$8.582\, e^{-(V+83.17)^2/786.6} + \frac{0.0269}{1+e^{(V-9.195)/2.982}} + 0.1886$
τ_{h2}	$1097\, e^{-(V+87.77)^2/377.4} + 2.987$	$320.3\, e^{-(V+87.77)^2/377.4} + 0.872$
G_K	4.664 mS/cm^2	6.609 mS/cm^2
n_{ss}	$\frac{1}{1+e^{(V+71.88)/14.32}}$	$\frac{1}{1+e^{(V+80.13)/14.32}}$
τ_{n1}	$0.1927\, e^{-(V+106.8)^2/390.5} + \frac{1.588}{1+e^{(V-9.861)/983.1}} - 0.6923$	$0.1181\, e^{-(V+106.8)^2/390.5} + \frac{0.9782}{1+e^{(V-9.861)/983.1}} - 0.4241$
τ_{n2}	$1.628\, e^{-(V+96.75)^2/399.6} + \frac{2.057}{1+e^{(V+92.94)/32.09}} + 0.0521$	$0.9973\, e^{-(V+96.75)^2/399.6} + \frac{1.260}{1+e^{(V+92.94)/32.09}} + 0.0319$
$G_{Na,\,wt}$	0.22 mS/cm^2 *	0.6976 mS/cm^2
o_{ss}	$\frac{1}{1+e^{(V+46.25)/-10.59}}$	$\frac{1}{1+e^{(V+46.25)/-10.59}}$
τ_o	0.2980	0.0797
p_{ss}	$\frac{0.9292}{1+e^{(V+64.67)/8.738}} + 0.0708$	$\frac{0.9292}{1+e^{(V+58.91)/8.738}} + 0.0708$
τ_p	0.9953	0.2663
$G_{K,\,wt}$	0.1313 mS/cm^2 *	0.1332 mS/cm^2
b_{ss}	$\frac{1}{1+e^{(V+9.055)/-23.79}}$	$\frac{1}{1+e^{(V+24.96)/-36.58}}$
τ_b	1.60	0.5840

* Starred current density values taken from literature, not from experimental Ex293 cultures; therefore, change in current density of wild type currents between 23C and 35C not indicative of true temperature-dependent current density change
